# SAGA DUBm-mediated surveillance regulates prompt export of stress-inducible transcripts for proteostasis

**DOI:** 10.1038/s41467-019-10350-6

**Published:** 2019-06-05

**Authors:** Minhoo Kim, Yoonjung Choi, Harim Kim, Daeyoup Lee

**Affiliations:** Department of Biological Sciences, Korea Advanced Institute of Science and Technology, Daejeon, 34141 South Korea

**Keywords:** RNA transport, Gene expression, Gene regulation, Chromatin remodelling, Proteasome

## Abstract

During stress, prompt export of stress-inducible transcripts is critical for cell survival. Here, we characterize a function of the SAGA (Spt-Ada-Gcn5 acetyltransferase) deubiquitylating module (DUBm) in monitoring messenger ribonucleoprotein (mRNP) biogenesis to regulate non-canonical mRNA export of stress-inducible transcripts. Our genetic and biochemical analyses suggest that there is a functional relationship between Sgf73p of DUBm and the essential mRNA export factor, Yra1p. Under physiological conditions, Sgf73p is critical for the proper chromatin localization and RNA binding of Yra1p, while also quality controlling the biogenesis of mRNPs in conjunction with the nuclear exosome exonuclease, Rrp6p. Under environmental stress, when immediate transport of stress-inducible transcripts is imperative, Sgf73p facilitates the bypass of canonical surveillance and promotes the timely export of necessary transcripts. Overall, our results show that the Sgf73p-mediated plasticity of gene expression is important for the ability of cells to tolerate stress and regulate proteostasis to survive under environmental uncertainty.

## Abstract (web summary)

Stress-inducible transcripts are quickly exported to preserve cell survival when cells are under stress. Here, the authors suggest that Sgf73p of the SAGA deubiquitylating module monitors messenger ribonucleoprotein biogenesis to regulate non-canonical export of stress-inducible transcripts.

## Introduction

In the eukaryotic cell, the genetic information is compartmentalized into a structure called the nucleus, enabling the temporal and spatial regulation of gene expression. Gene expression involves various steps, ranging from transcriptional activation, elongation, and RNA processing in the nucleus, to the transport of export-competent mRNAs to the cytoplasm for translation. During the tightly coordinated process of mRNA export, various factors are recruited to the transcription site and nascent RNA. The TRanscription and EXport (TREX) complex consists of the THO complex and Yra1p/Sub2p, which are involved in transcription elongation and mRNA export, respectively^1,2^. Yra1p is responsible for recruiting the Mex67p-Mtr2p complex, which binds to the newly synthesized mRNA and escorts it to the nuclear pore complex (NPC)^3,4^. Finally, a physical interaction between Mex67p and the TREX-2-NPC complex facilitates the export of properly assembled messenger ribonucleoproteins (mRNPs) to the cytoplasm^5,6^.

Under physiological conditions, surveillance systems strictly ensure that only export-competent mRNPs are transported. In yeast, mRNP quality control is monitored by Rrp6p, the exonuclease of the nuclear exosome complex^7,8^. Studies have shown that Rrp6p is required for the nuclear retention of aberrant mRNPs in 3′-end processing-defective mutants^8,9^. In contrast, upon environmental stress, the canonical mRNA export process is bypassed and immediate export of stress-related transcripts is undertaken to maintain proteostasis and maximize cell survival.

We previously reported that a physical interaction between the proteasomal 19S regulatory particle (RP) and the SAGA complex (a highly conserved transcriptional co-activator) is important for mRNA export in *Saccharomyces cerevisiae*^10^. The proteasome–SAGA interaction causes a functional Sgf73-DUBm (deubiquitylating module) subcomplex to separate from SAGA. The *rpt2-1* mutant, which shows perturbation of the proteasome–SAGA interaction, also exhibits significant retention of Sgf73-DUBm near promoter regions, along with defects in mRNA export.

In the present work, we characterize a functional relationship between Sgf73p of the DUBm and the essential mRNA export factor, Yra1p. Under normal conditions, Sgf73p directly interacts with Yra1p and is important for its proper chromatin localization and transcript binding. Here, we demonstrate that Sgf73p plays a central role in the surveillance of mRNP biogenesis through a concerted action with Rrp6p. During environmental stress, when prompt export of stress-related transcripts is required, Sgf73p promotes a bypass of the canonical mRNA export surveillance and allows immediate export of specific transcripts, including those of molecular chaperone genes. This facilitates cellular proteostasis by acting to unfold protein aggregates in the cytoplasm. Our results collectively show that, in response to a stressful environmental change, Sgf73p orchestrates the selective export of immediate genes by occupying their upstream-activating sequences (UASs), and is thus crucial to sustaining cell adaptability.

## Results

### *sgf73∆* restores the growth defects of mRNA export mutants

**Fig. 1:**
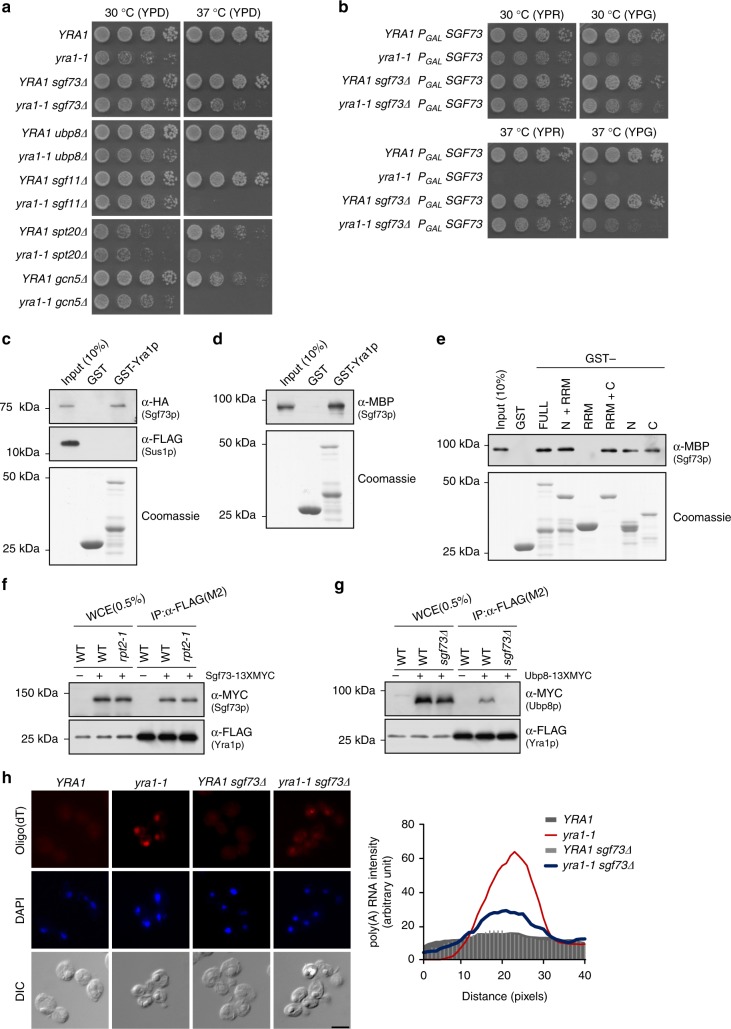
Functional relationship between Sgf73p and Yra1p. **a** Spotting assays to assess genetic interactions between *yra1-1* and SAGA subunit-deletion mutants. Cells were spotted onto YPD plates with five-fold serial dilutions and incubated at 30 and 37 °C. **b** Spotting assays to assess the effects of Sgf73p overexpression in the *yra1-1* mutant. Cells transformed with *P*_*GAL*_ *SGF73* inserted into the *URA3* locus were cultured in YP + 2% raffinose (YPR) medium and plated on YPR and YPG (YP + 2% galactose) media. Plates were incubated at 30 and 37 °C. **c** In vitro pulldown assay of gel-filtration-purified Sgf73-DUBm against GST-tagged Yra1p. Binding was assessed by Western blotting against Sgf73p (α-HA) and Sus1p (α-FLAG). **d** In vitro pulldown assay of recombinant Sgf73p against GST-tagged Yra1p. Binding was assessed by Western blotting against Sgf73p (α-MBP). **e** In vitro pulldown assays were performed using Sgf73p (α-MBP) and the indicated subfragments of Yra1p (α-GST). **f** Co-immunoprecipitation (co-IP) assays of Sgf73p (α-13xMYC) and Yra1p (α-5xFLAG) in wildtype and *rpt2-1* cells. Yra1p (α-5xFLAG) was immobilized on anti-FLAG M2 affinity gel and co-IP was assessed by Western blotting. **g** Co-IP assay of Ubp8p (α-13xMYC) and Yra1p (α-5xFLAG) in wildtype and *sgf73Δ* cells. Yra1p (α-5xFLAG) was immobilized on anti-FLAG M2 affinity gel and co-IP was assessed by Western blotting. **h** (Left panel) FISH analyses of YRA1, *yra1-1*, YRA1 *sgf73Δ*, and *yra1-1 sgf73Δ*. Cells were cultured at 30 °C to early log-phase and shifted to 37 °C for 2 h. Poly-(A) + RNA was detected using Cy3-labeled oligo(dT) probes and DNA was counterstained with 4′,6-diamidino-2-phenylindole (DAPI). Scale bar, 5 μm. (Right panel) Quantification graph of poly-(A) + RNA intensity. Poly-(A) + RNA intensity was quantified using the same scan width of respective nuclei (determined by DAPI staining). WCE, whole-cell extract. Coomassie blue staining was used as a loading control

To investigate how the retention of Sgf73p affects mRNA export, we assessed whether there was a genetic interaction between *SGF73* and *YRA1*. The essential mRNA export factor, Yra1p, is recruited as part of the TREX complex and mediates the association of the Mex67p–Mtr2p complex with nascent transcripts^1,2,11^. Surprisingly, at a non-permissive temperature (37 °C), *SGF73* deletion partially restored the growth defect seen in the *yra1-1* mutant^11^ (Fig. 1a, upper panel), whereas deletion of two other subunits of the Sgf73-DUBm, *UBP8*, or *SGF11*, did not (Fig. 1a, middle panel). The deletion of *SUS1*, which is another subunit of Sgf73-DUBm, was reportedly associated with synthetic lethality when combined with *yra1-1*, possibly indicating that Sus1p plays a critical role in mRNA export as a component of the TREX-2 complex^12^. Removal of Gcn5p, the acetyltransferase of SAGA, did not affect the growth defect of *yra1-1*, but deletion of *SPT20*, which causes disintegration of the entire SAGA complex, partially restored *yra1-1* cell growth (Fig. 1a, lower panel). To test whether the observed genetic interaction was restricted to the *yra1-1* mutant, we combined *SGF73* deletion with a number of well-characterized mRNA export-defective mutants, including *mex67-5*, *mtr2-9, sub2-*85, and *npl3∆* (Supplementary Fig. 1a–d). Indeed, the loss of *SGF73* partially restored cell growth in all tested mRNA export-defective mutants. These results indicate that deletion of *SGF73* improves the overall fitness of cells growing in a mRNA export-defective environment. Interestingly, *SGF73* did not rescue the growth defect of *nab2-34*, another mRNA export-defective mutant. Npl3p and Nab2p were reported to function as independent adapters of the mRNA export factor, Mex67p, suggesting that Sgf73p and Nab2p play distinct roles^13^.

To further confirm the positive genetic interaction between *SGF73* and *YRA1*, we used the galactose-inducible promoter, *P*_GAL_, to overexpress Sgf73p in certain mutants (Fig. 1b and Supplementary Fig. 1f). We observed notable suppression of the rescue phenotype in the *yra1-1 sgf73∆* mutant at the restrictive temperature upon Sgf73p overexpression (Fig. 1b, lower panel, YPG), while a lesser extent of rescue was seen at the permissive temperature (30 °C) (Fig. 1b, upper panel, YPG). Interestingly, overexpression of Sgf73p inhibited the growth of even *yra1-1-*mutant cells incubated at the permissive temperature.

To assess potential differences in the gene expression patterns of the *SGF73* deletion mutant compared to wildtype, we generated and analyzed mRNA-seq data. As shown in Supplementary Fig. 2, we did not observe any significant change in transcription for the *sgf73∆* mutant. Overall, our findings show that Sgf73p has a function in mRNA export that is independent of its function in transcriptional regulation.

### Yra1p is a bona fide binding partner of Sgf73p

Given the positive genetic interaction between *SGF73* and *YRA1*, we speculated that these factors may physically interact. Indeed, our in vitro GST-protein pulldown assays indicated that both gel-filtration-purified Sgf73-DUBm (Fig. 1c) and recombinant Sgf73p (Fig. 1d) physically interacted with Yra1p. Interestingly, the Sgf73-DUBm subunit, Sus1p, was not recovered in the immunoprecipitated fraction (Fig. 1c, middle panel), suggesting that Sus1p has a different fate after Yra1p binding. Sus1p is a subunit of the TREX-2 complex, whose interaction with the NPC is essential for proper mRNA export^14^; indeed, Sgf73p has been shown to be important for proper assembly of the TREX-2 complex^15^.

Yra1p is composed of an RNA-recognition motif (RRM), two highly conserved N-terminal and C-terminal RNA export factor-binding protein (REF) domains, and two moderately conserved regions that flank the RRM, called the N-variable and C-variable domains^3,11^. To further define the region(s) within Yra1p responsible for the physical interaction with Sgf73p, we generated subfragments of Yra1p using previously published GST-Yra1p constructs^16^ (Supplementary Fig. 3a). We found that Sgf73p bound to the N-terminal and C-terminal regions of Yra1p, but not the RRM (Fig. 1e). Surprisingly, Pcf11p, a component of the cleavage and polyadenylation complex, was previously reported to bind the same domain within Yra1p^16,17^.

To confirm the physical interaction between Sgf73p and Yra1p in vivo, we performed co-immunoprecipitation (co-IP) assays. Yra1p showed binding with Sgf73p in both wildtype and *rpt2-1* cells (Fig. 1f). As the separation of the Sgf73-DUBm is inhibited in the *rpt2-1* mutant, the binding of Yra1p with Sgf73p in the mutant indicates that such binding occurs when Sgf73p is intact within the whole SAGA complex. Ubp8p showed binding with Yra1p in wildtype cells but not in *sgf73∆* mutant cells, supporting the direct interaction between Sgf73p and Yra1p (Fig. 1g). Ada1p and Spt20p, which are two additional subunits of the SAGA complex, interacted with Yra1p in wildtype cells, but this association was significantly reduced in *sgf73∆* cells (Supplementary Fig. 3b, c). Yra1p has been demonstrated to be recruited to transcribed regions by the RNAPII C-terminal domain (CTD)-bound 3′-end processing factor, Pcf11p^16^. However, Sgf73p did not show any physical interaction with Pcf11p in vivo (Supplementary Fig. 3d), and thus is unlikely to be involved in the initial recruitment of Yra1p. In contrast, Sub2p, whose binding with Yra1p is reported to occur after chromatin recruitment of Yra1p, showed binding in vivo (Supplementary Fig. 3e). From these findings, we hypothesize that Sgf73p interacts with Pcf11p-bound Yra1p to enable subsequent processes of the mRNA export pathway.

To further assess the functional relationship between Sgf73p and Yra1p in regulating mRNA export, we performed fluorescence in situ hybridization (FISH). As previously reported, a severe mRNA export defect was observed in the *yra1-1* mutant at the non-permissive temperature (37 °C). Consistent with the findings from our genetic interaction assays, the mRNA export defects in *yra1-1* cells were rescued by Sgf73p deletion (Fig. 1h). Taken together, these findings indicate that Sgf73p contributes to nuclear mRNA retention through Yra1p.

### Sgf73p acts as an RNA surveillance factor during mRNA export

**Fig. 2:**
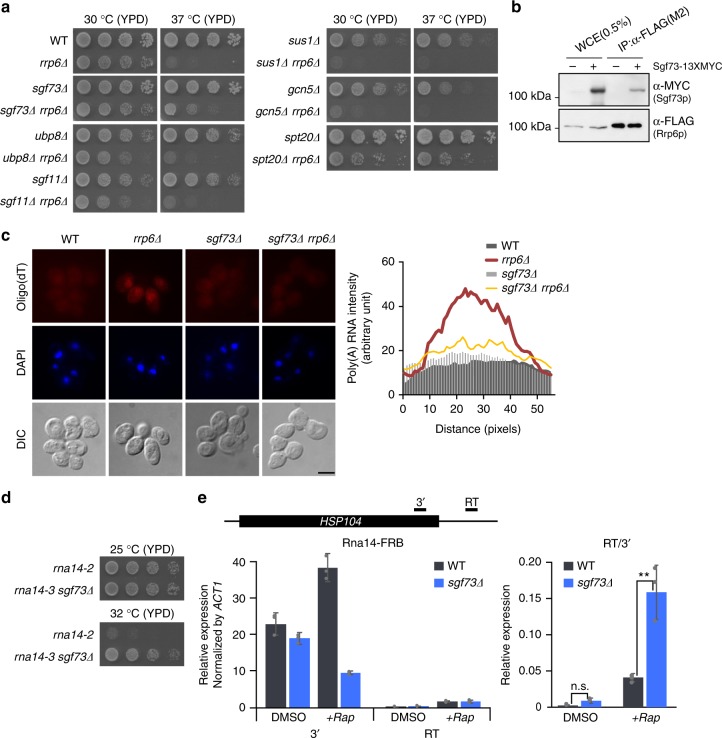
Sgf73p functions in mRNP quality control. **a** Spotting assays to assess genetic interactions between *rrp6Δ* and SAGA subunit-deletion mutants. Cells were spotted onto YPD plates with five-fold serial dilutions and incubated at 30 and 37 °C. **b** Co-IP assay of Sgf73p (α-MYC) and Rrp6p (α-FLAG). Rrp6p (α-5xFLAG) was immobilized on anti-FLAG M2 affinity gel and co-IP was assessed by Western blotting. **c** (Left panel) FISH analyses of wildtype, *rrp6Δ*, *sgf73Δ*, and *sgf73Δrrp6Δ* cells. Poly-(A) + RNA was detected using Cy3-labeled oligo(dT) probes and DNA was counterstained with DAPI. Scale bar, 5 μm. (Right panel) Quantification graph of poly-(A) + RNA intensity. Poly-(A) + RNA intensity was quantified using the same scan width of respective nuclei (determined by DAPI staining). **d** Spotting assays to assess the genetic interaction between *rna14-3* and *sgf73Δ*. Cells were spotted onto YPD plates with five-fold serial dilutions and incubated at 25 and 32 °C. **e** Quantification of read-through transcripts from the *HSP104* gene in wildtype and *sgf73Δ* cells. Wildtype and *sgf73Δ* cells were depleted of Rna14p using the anchor-away method (*+Rap*). Levels of read-through transcripts were normalized by 3′-transcripts. Standard deviations of three independent experiments are shown by error bars and *P*-values were determined by Student’s *t*-test (*P ≤ 0.05, **P ≤ 0.01, and ***P ≤ 0.001). WCE, whole cell extract. RT, read-through

The involvement of Sgf73p in the nuclear retention of mRNAs suggests that it may contribute to quality control during mRNP biogenesis. The nuclear exosome, Rrp6p, is reported to be the key factor in the surveillance of mRNP assembly and export^8^. Thus, we speculated that, as a quality control factor, Sgf73p is likely to display a functional relationship with Rrp6p. Interestingly, we found that Sgf73p deletion partially rescued the growth defect of the *rrp6∆* mutant at the restrictive temperature. Consistent with the genetic interactions observed between Yra1p and the SAGA complex, the positive interaction with the *rrp6∆* mutant was restricted to *SGF73* within the DUBm (Fig. 2a). A rescue phenotype was also observed between the *spt20∆* and *rrp6∆* mutants. A negative genetic interaction between *RRP6* and *SUS1* was previously observed through high-throughput analysis^18^, and depletion of *RRP6* was reported to yield synthetic lethality when combined with mRNA export mutants, including *YRA1*^19^. Additionally, a physical interaction between Sgf73p and Rrp6p in vivo was confirmed by co-IP assays (Fig. 2b). To test whether they have a cooperative function, we assessed mRNA export defects in the respective mutants. We observed significant retention of transcripts in the *rrp6∆* mutant, and found that this nuclear retention of mRNAs was alleviated by the deletion of *SGF73* (Fig. 2c).

The identified functional relationship between Sgf73p and Rrp6p prompted us to assess how Sgf73p deletion affects a 3′-end processing-defective mutant. *RRP6* deletion was reported to rescue the growth defects of 3′-end processing-defective mutants, including *rna14-3*^20^. Similarly, we found that the growth of *rna14-3* cells was restored by deletion of *SGF73* (Fig. 2d).

Under normal conditions, only export-competent transcripts are stringently exported to the cytoplasm for further processes of gene expression; in mRNA export-defective or 3′-end processing-defective mutants, there are severe growth defects due to nuclear retention of transcripts that are necessary for normal cellular functions. In the absence of a functional surveillance system, such as in the *SGF73* or *RRP6* deletion mutants, premature transcripts can bypass quality control and undergo nucleocytoplasmic transport. Supply of premature transcripts is reportedly less detrimental to cells than their complete absence^21^. Deletion of *RRP6* was shown to improve the cell fitness of the 3′-end processing-defective mutant, *rna14-3*^22^. Thus, despite the improvement in overall cell growth, we hypothesized that certain 3′-end formation-defective phenotypes would be amplified in the absence of the surveillance factor, Sgf73p. To test this, we used a previously reported system in which changes in the amount of read-through transcripts produced from the *HSP104* gene are measured in the *rna14-3* mutant background^23^. We depleted cells of Rna14p using the anchor-away method (Rna14-AA)^24^. We detected significantly more read-through transcripts in Rna14-AA cells exposed to rapamycin (*+Rap*) compared to those treated with DMSO (Fig. 2e, left panel). Moreover, the amount of read-through transcripts was significantly increased in the *sgf73∆* Rna14-AA mutant (Fig. 2e, right panel), suggesting that 3′-end processing defects are exacerbated by the deletion of *SGF73*.

Overall, our findings support a model in which the concerted actions of Sgf73p and Rrp6p ensure proper quality control of nuclear mRNP biogenesis.

### Identification of the Sgf73p-regulated gene set

**Fig. 3:**
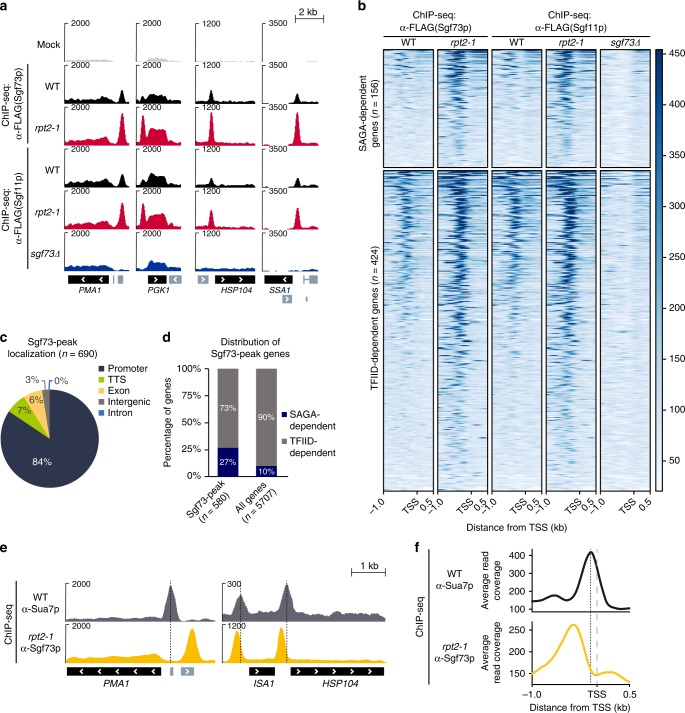
Sgf73p is globally retained in the UAS elements of genes in *rpt2-1*. **a** Genome browser view of the *PMA1*, *PGK1*, *HSP104*, and *SSA1* genes showing the ChIP-seq profiles of Sgf73p and Sgf11p in mock, wildtype, *rpt2-1*, and *sgf73Δ* cells. **b** Heatmaps of ChIP-seq signals representing Sgf73-DUBm binding (α-Sgf73p and α-Sgf11p) in wildtype, *rpt2-1*, and *sgf73Δ* cells 1 kb upstream and 0.5 kb downstream of the TSS in the Sgf73-peak gene set (*n* = 580). Heatmaps were divided into SAGA-dependent genes (*n* = 156) and TFIID-dependent genes (*n* = 424) and sorted in descending order of Sgf73p enrichment. The *y*-axis color scale indicates normalized ChIP-seq read count. **c** Pie chart showing the Sgf73-peak localization pattern. **d** Bar graph indicating the distribution of Sgf73-peak genes within the SAGA-dependent genes (73%) and TFIID-dependent genes (27%). **e** Genome browser view of the *PMA1*, *ISA1*, and *HSP104* genes showing the ChIP-seq profiles of Sua7p in wildtype and Sgf73p in *rpt2-1* cells. Dotted lines indicate Sua7p peak sites. **f** Average plots of ChIP-seq signals representing the binding of Sua7p and Sgf73p in wildtype and *rpt2-1* cells. Dashed and dotted lines indicate the TSS and Sua7p-binding sites, respectively. kb, kilobase. TSS, transcription start site

We previously reported a significant retention of Sgf73p-DUBm near promoter regions in the proteasome–SAGA interaction-defective mutant, *rpt2-1*^10^. To investigate the genes that are directly influenced by Sgf73p retention, we performed chromatin immunoprecipitation followed by high-throughput sequencing (ChIP-seq) against 5xFLAG-tagged Sgf73p. We confirmed the strong accumulation of Sgf73p in the *rpt2-1* mutant near promoter regions of the representative genes used in our previous report (*PMA1* and *PGK1*), as well as those of other genes, including *HSP104* and *SSA1* (Fig. 3a). As shown in Fig. 3b, Sgf73-DUBm was retained near promoter regions throughout the genome in *rpt2-1*. To further confirm this localization, we performed ChIP-seq analysis against another subunit of the Sgf73-DUBm, Sgf11p. Indeed, Sgf11p showed co-localization with Sgf73p in *rpt2-1* cells, whereas in the *sgf73∆* mutant, which entirely lacks the DUBm in its SAGA complex, Sgf11p showed a substantial, if not complete, decrease in enrichment (Fig. 3a, b).

By using HOMER^25^ to perform peak-finding analysis, we identified and annotated genes with significant Sgf73-DUBm peaks (*n* = 690). Among the identified peaks, 84% (*n* = 580) were found near promoter regions (Fig. 3c). For our further analyses, we focused on the genes that exhibited Sgf73-DUBm peaks near their promoter region, which we herein refer to as Sgf73-peak genes. To compare the localization of Sgf73-DUBm with that of SAGA, we utilized recently published ChEC-seq data for several SAGA subunits^26^. The ChEC-seq signals against Spt7p and Ubp8p showed enrichment in genes with Sgf73-DUBm peaks (*n* = 580) (Supplementary Fig. 4a). We used HOMER to identify and annotate genes with Spt7p (*n* = 4366) and Ubp8p (*n* = 4770) peaks, and found that the Sgf73-peak genes showed significant overlaps with the Spt7-peak and Ubp8-peak genes (*P*-value = 1e−12) (Supplementary Fig. 4b).

To further characterize the Sgf73-DUBm peaks observed in the *rpt2-1* mutant, we aligned Sgf73-DUBm enrichment with core promoters, marked by TFIIB (encoded by the *SUA7* gene) occupancy. Interestingly, the Sgf73-DUBm peaks were enriched in regions upstream of core promoters (Fig. 3e, f). As UASs are reportedly located 60–300 bp upstream of core promoters^27^, we speculate that Sgf73-DUBm is likely to be recruited to UASs as part of the full SAGA complex, and that Sgf73-DUBm is subsequently separated by the proteasome for further downstream regulation.

SAGA has been implicated in regulating stress-inducible genes, whereas TFIID is believed to be responsible for controlling the expression of housekeeping genes^28,29^. However, there has been some controversy regarding this division of function, as recent reports suggest that the SAGA complex plays a broader role in regulating gene expression^26,30^. Interestingly, although the Sgf73-DUBm peak genes identified in the present work did not show any particular tendency towards the SAGA-dependent or TFIID-dependent gene groups (Fig. 3b, d), we observed sharp Sgf73-DUBm peaks in some well-characterized stress-inducible genes, including heat shock genes (e.g., *HSP104* and *SSA1*) (Fig. 3a). Based on these findings, we are convinced that the previous attempts to classify SAGA-regulated genes by simply depleting SAGA subunits and performing steady-state transcriptomic analysis were insufficient to fully reflect the regulatory dependency of genes. Rather, it seems likely that SAGA per se orchestrates the expression of various gene sets via a more dynamic mode of regulation.

### Sgf73p is required for proper chromatin recruitment of Yra1p

**Fig. 4:**
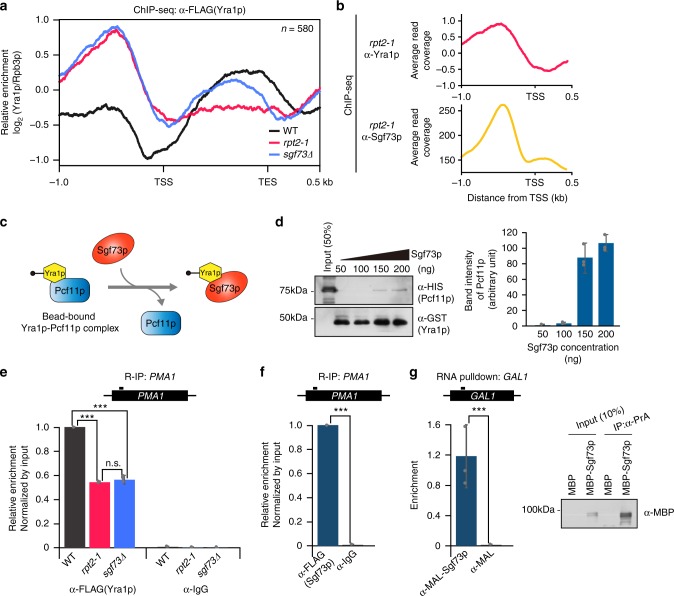
Sgf73p participates in transitioning Yra1p onto nascent transcripts. **a** Average plots of ChIP-seq signals representing the binding of Yra1p in wildtype, *rpt2-1* and *sgf73Δ* cells from 1.0 kb upstream of the TSS to 0.5 kb downstream of the TES, regarding the Sgf73-peak gene set (*n* = 580). **b** Average plots of ChIP-seq signals representing the binding of Yra1p and Sgf73p in the *rpt2-1* mutant. **c** Schematic diagram of in vitro competition assay between Sgf73p and Pcf11p with respect to Yra1p binding. The Yra1p-Pcf11p complex was immobilized on GST sepharose beads. After a brief wash, Sgf73p was added and the amount of released Pcf11p in supernatants was assessed by Western blotting. **d** (Left panel) Western blotting of an in vitro competition assay performed between Sgf73p and Pcf11p (α-HIS) with respect to the binding of Yra1p (α-GST). (Right panel) Band intensity of released Pcf11p in the supernatant following the addition of Sgf73p. **e** R-IP-qPCR analysis of Yra1p (α-FLAG) and control (α-IgG) against the *PMA1* transcript in wildtype, *rpt2-1*, and *sgf73Δ* cells. **f** R-IP-qPCR analysis of Sgf73p (α-FLAG) and control (α-IgG) against the *PMA1* transcript in wildtype cells. **g** (Left panel) In vitro RNA-pulldown analysis of Sgf73p (α-MBP) against the *GAL1* transcript. (Right panel) Western blotting against bead-bound Sgf73p (α-MBP). Standard deviations of all quantification data were obtained from three independent experiments and are shown by error bars; *P*-values were evaluated by Student’s *t*-test (*P ≤ 0.05, **P ≤ 0.01, and ***P ≤ 0.001). kb, kilobase. TSS, transcription start site. TES, transcription end site

The physical interaction between Sgf73p and Yra1p prompted us to use ChIP-seq analysis to assess the chromatin localization of Yra1p regarding the Sgf73-peak gene set. In wildtype cells, Yra1p showed a gradual increase in enrichment throughout the gene-body region, as previously reported (Fig. 4a)^31–33^. In the *rpt2-1* mutant, Yra1p was absent in the gene-body region and showed colocalization with Sgf73p in the UAS, confirming the physical interaction between the two factors (Fig. 4b). Interestingly, in the absence of Sgf73p, Yra1p was also retained in the UAS and showed slightly reduced occupancy throughout the gene-body (Fig. 4a). Since no physical interaction was observed between Sgf73p and Pcf11p in vivo and Pcf11p enrichment was not significantly altered in the mutants (Supplementary Fig. 5a, b), we believe that Sgf73p is not likely to be directly responsible for the initial recruitment of Yra1p. Additionally, although the tagging of Yra1p with large epitopes (e.g., GFP) was reported to result in growth defects, we did not detect any noticeable growth defect due to the tagging of Yra1p under our experimental conditions (Supplementary Fig. 5c, d).

Having observed that Sgf73p binds the same domain within Yra1p that also binds Pcf11p (Fig. 1e), we speculated that there could be competitive binding among Yra1p, Sgf73p, and Pcf11p. To further characterize the interaction among Yra1p, Sgf73p, and Pcf11p, we performed a competition assay in vitro (Fig. 4c). First, the Yra1p–Pcf11p complex was assembled on immobilized GST beads. The bead-bound complexes were washed briefly, Sgf73p was added and the amount of Pcf11p released to the supernatant was assessed by Western blotting. As shown in Fig. 4d, the amount of released Pcf11p gradually increased following the addition of Sgf73p. From these findings, we propose that Yra1p is handed over from Pcf11p to Sgf73p for further regulation (see the section “Discussion” for details).

### Yra1p loading onto nascent RNA transcripts requires Sgf73p

After its recruitment onto chromatin, Yra1p recruits the essential mRNA export factors, Mex67p-Mtr2p, and is loaded onto nascent RNA transcripts along with other factors for subsequent steps of the mRNA export process^3,4,34^. However, the molecular mechanism responsible for transitioning Yra1p from the transcriptional machinery to the nascent RNA is not yet fully understood.

Our Yra1p ChIP-seq analysis showed that Yra1p is significantly retained in the UAS and its enrichment along the gene-body is decreased in the *sgf73∆* mutant compared to wildtype (Fig. 4a). Based on this data, we hypothesized that the loading of Yra1p onto RNA is likely to be affected in this mutant. To analyze the changes in the RNA binding of Yra1p, we performed in vivo RNA-immunoprecipitation (R-IP) assays for the *PMA1* transcript in wildtype, *rpt2-1*, and *sgf73∆* cells. Yra1p showed significantly reduced RNA-binding activity in the *rpt2-1* mutant compared to wildtype (Fig. 4e), which likely reflects the retention of Yra1p in the UAS. Surprisingly, the RNA binding of Yra1p was reduced in the *SGF73* deletion cells, to a level similar to that of *rpt2-1*. Given that enrichment of Yra1p was observed throughout the gene-body in *sgf73∆* cells (Fig. 4a, *sgf73∆*), the reduced R-IP signals indicate that the Yra1p–RNA interaction is defective in the *sgf73∆* mutant. Similar patterns were observed for other representative transcripts, including those of *PGK1*, *HSP104*, and *SSA1* (Supplementary Fig. 6a–c).

As the findings described above suggest that Sgf73p plays a role in transitioning Yra1p onto RNA transcripts, we hypothesized that this could occur via the direct binding of RNA by Sgf73p. Our R-IP assays revealed that Sgf73p has a strong RNA-binding activity (Fig. 4f and Supplementary Fig. 6). To further confirm the RNA-binding activity of Sgf73p, we performed an in vitro RNA pulldown assay and RNA electrophoretic mobility shift assay (RNA-EMSA) using the *GAL1* transcript. Consistent with our in vivo data, Sgf73p showed significant RNA binding in vitro (Supplementary Fig. 6d). Collectively, these findings indicate that Sgf73p is required for the proper loading of Yra1p onto nascent transcripts, possibly through its direct binding with RNA.

### Sgf73p specifically occupies the UASs of inducible genes

**Fig. 5:**
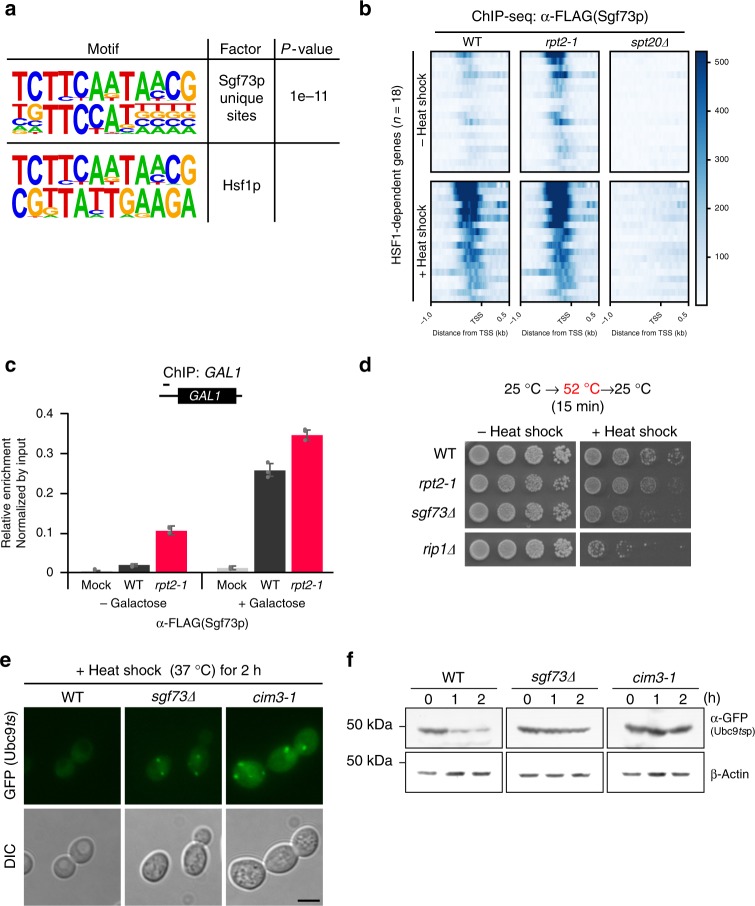
Sgf73p is required for heat shock survival and proteostasis. **a** (Upper panel) De novo motif enrichment analysis of Sgf73p-binding sites was performed using HOMER. (Lower panel) Comparison of the Sgf73p-binding motif with known motifs showed significant correspondence with the Hsf1p-binding motif. **b** Heatmaps of ChIP-seq signals representing Sgf73p-binding (α-FLAG) in wildtype, *rpt2-1*, and *spt20Δ* cells, from 1 kb upstream to 0.5 kb downstream of the TSS regarding Hsf1-dependent genes (*n* = 18) before and after heat shock. Heatmaps were sorted in descending order of Sgf73p enrichment. The *y*-axis color scale indicates normalized ChIP-seq read count. **c** ChIP-qPCR analysis against Sgf73p (α-FLAG) of the *GAL1* gene before and after galactose induction in mock, wildtype, and *rpt2-1* cells. **d** Cell survival analysis after heat shock was assessed by spotting assays. Wildtype, *rpt2-1*, and *sgf73Δ* cells were subjected to heat shock at 52 °C for 15 min, plated on YPD medium in five-fold serial dilutions, and incubated at 25 °C for 3 days. **e** Visualization of GFP-Ubc9*ts* by fluorescence imaging. Wildtype, *rpt2-1* and *sgf73Δ* cells carrying the galactose-inducible GFP-Ubc9ts construct were subjected to heat shock at 37 °C for 2 h, and then fixed and imaged. Scale bar, 5 μm. **f** GFP-Ubcp9*ts* protein levels (α-GFP) in wildtype, *rpt2-1*, and *sgf73Δ* cells subjected to heat shock were assessed by Western blotting. β-Actin was used as a loading control. Standard deviations of three independent experiments are shown by error bars. kb, kilobase. TSS, transcription start site

Motif analysis using HOMER indicated that there was a strong correlation between the binding sequences of Sgf73p and Hsf1p (Fig. 5a). A recent study stringently identified genes that are directly regulated by yeast heat shock factor 1 (Hsf1) upon stress^35^. Consistent with the motif analysis, Sgf73-DUBm peaks were found in the UASs of all 18 Hsf1-regulated genes in the *rpt2-1* mutant (Supplementary Fig. 7a).

To investigate a possible stress-related function of Sgf73p, we analyzed the changes in Sgf73p retention during heat shock with respect to the Hsf1-dependent genes. Upon heat shock, we observed a significant increase in the enrichment of Sgf73p at the Hsf1-dependent gene set (*n* = 18) in wildtype cells (Fig. 5b). Interestingly, the intensity of Sgf73p retention was similar between wildtype and *rpt2-1* cells after heat shock. The Hsf1-dependent genes were previously shown to be required for cell survival following other environment changes, including oxidative stress^36^. To test whether the enrichment of Sgf73p is heat shock-specific, we performed ChIP-qPCR against 5xFLAG-tagged Sgf73p under oxidative stress. Indeed, the Sgf73p peak increased in wildtype cells, to a level similar to that seen in *rpt2-1* cells (Supplementary Fig. 8a, b).

In an effort to characterize a global function of Sgf73p retention, we assessed another inducible gene, *GAL1*. Using ChIP-qPCR, we observed significant retention of Sgf73p (approximately five-fold the wildtype level) within the *GAL1* UAS of the non-induced *rpt2-1* mutant (Fig. 5c, −Galactose). Surprisingly, galactose induction increased the occupancy of Sgf73p in wildtype cells to a level similar to that seen in *rpt2-1* cells (Fig. 5c, +Galactose). To confirm the causal relationship between galactose induction and the increased retention of Sgf73p in wildtype cells, we assessed Sgf73p occupancy near the promoter regions of the *PMA1* and *SSA1* genes, whose expression levels should be minimally affected by galactose induction (Supplementary Fig. 8c). Our ChIP-qPCR results revealed that, in contrast to the *GAL1* gene, the enrichment of Sgf73p at *PMA1* and *SSA1* genes differed significantly between wildtype and *rpt2-1* cells. Thus, the increased Sgf73p enrichment at the UAS of the *GAL1* gene appears to be a specific response to galactose induction.

### Sgf73p plays a key role in proteostasis

To investigate the physiological consequences of Sgf73p retention during stress tolerance, we assessed the cell survival rate upon heat shock. Wildtype, *rpt2-1* and *sgf73∆* cells were subjected to severe heat shock (15 min at 52 °C), plated on rich medium and incubated at a physiological temperature. *sgf73∆* cells showed reduced cell survival upon heat shock (Fig. 5d). Moreover, we found that Sgf73p is required for heat shock survival in other isogenic backgrounds, including W303a and BY4741 (Supplementary Fig. 8d). Consistent with the above-described ChIP-qPCR results, the *sgf73∆* mutant showed reduced tolerance to oxidative stress and a decreased growth rate in galactose-based medium (Supplementary Fig. 8e, f).

Most of the Hsf1-dependent genes encode chaperone transcripts and thus are closely related to proteostasis. To directly observe the function of Sgf73p in proteostasis upon heat shock, we utilized the GFP-tagged Ubc9*ts* mutant protein. Upon heat shock, Ubc9*ts* proteins form distinctive foci that are eventually resolved by the ubiquitin–proteasome pathway in wildtype cells, whereas they are retained in proteostasis-compromised mutants^37,38^. After 2 h of heat shock, wildtype cells displayed complete elimination of Ubc9*ts* foci, whereas the proteolytic mutant, *cim3-1*, still displayed distinctive foci (Fig. 5e)^39^. Consistent with the findings described above, the *sgf73∆* mutant exhibited Ubc9*ts* foci similar to those found in the *cim3-1* mutant. To confirm the imaging data, we prepared whole-cell extracts from heat-shocked cells and used Western blotting to quantify the GFP-Ubc9*ts* protein levels. After 2 h of heat shock, the Sgf73p deletion mutant showed a significantly higher level of GFP-Ubc9*ts* proteins compared to wildtype cells (Fig. 5f).

Overall, these findings show that the retention of Sgf73p in the UAS of specific genes is required for stress tolerance and cell survival.

### Sgf73p retention promotes non-canonical mRNA export

**Fig. 6:**
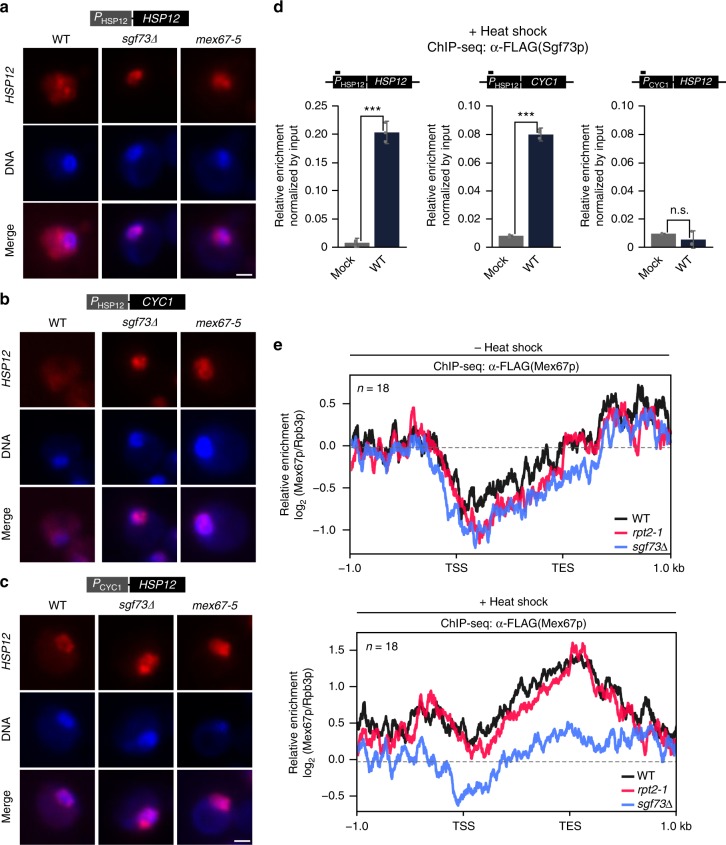
Sgf73p promotes export of specific transcripts during heat shock. **a**–**c** FISH analyses against (**a**) stress-inducible P_HSP12_ *HSP12*, (**b**) stress-inducible P_HSP12_ *CYC1*, and (**c**) non-inducible P_CYC1_ *HSP12* genes were performed using Cy3-labeled specific probes. DNA was counterstained with DAPI. Scale bar, 1 μm. **d** ChIP-qPCR analysis against Sgf73p (α-FLAG) in mock and wildtype cells after heat shock, regarding a stress-inducible promoter (left and middle panel) and a non-inducible promoter (right panel). **e** Average plot of Mex67-5xFLAG enrichment in wildtype, *rpt2-1*, and *sgf73Δ* cells with respect to the Hsf1-dependent gene set (*n* = 18) before and after heat shock. Standard deviations obtained from three independent experiments are shown by error bars and *P*-values were determined by Student’s *t*-test (*P ≤ 0.05, **P ≤ 0.01, and ***P ≤ 0.001)

Having shown that Sgf73p plays roles in proteostasis and the regulation of Hsf1-dependent genes during stress, we speculated that Sgf73p is likely to promote the efficient export of stress-inducible transcripts. To directly assess the possible function of Sgf73p in the export of Hsf1-dependent transcripts during stress, we performed single-molecule FISH (smFISH) against the stress-inducible *HSP12* RNA after heat shock. As shown in Fig. 6a, we observed significant retention of the *HSP12* transcript in the *sgf73∆* mutant, similar to that seen in *mex67-5*. ChIP-qPCR analysis confirmed that there was significant accumulation of Sgf73p on the inducible promoter/UAS *P*_*HSP12*_ upon heat shock in wildtype cells, whereas none was observed on the non-Hsf1p-dependent *P*_*CYC1*_ (Fig. 6d). When the promoter/UAS of the *CYC1* gene was replaced with that of *HSP12*, we observed proper *CYC1* transcript export and Sgf73p retention in wildtype cells (Fig. 6b–d), suggesting that Sgf73p accumulation is important for the export of stress-inducible transcripts upon heat shock. Additionally, we observed a physical interaction between Sgf73p and Hsf1p in vivo (Supplementary Fig. 9a). Together, our data show that Sgf73p plays an essential role in the specific export of Hsf1-dependent transcripts during stress.

A recent report demonstrated that Mex67p alone has an independent function in the immediate mRNA export of certain transcripts, such as those encoding chaperones, during heat shock^40^. The same study showed that, during this process, canonical mRNA export factors are absent from the transcripts and quality control is bypassed for timely export. We performed ChIP-seq analysis against 5xFLAG-tagged Mex67p in wildtype, *rpt2-1*, and *sgf73∆* cells before and after heat shock (Fig. 6e). Interestingly, the levels of Mex67p were similar in wildtype, *rpt2-1*, and *sgf73∆* cells under normal growth conditions, whereas Mex67p enrichment was significantly reduced in the *sgf73∆* mutant under heat shock. Additionally, the enrichment of Mex67p at non-Hsf1p genes did not significantly differ in wildtype, *rpt2-1*, and *sgf73∆* cells (Supplementary Fig. 9b, c). Based on these data, we propose the following model: During environmental stress, such as heat shock, the retention of Sgf73p at UASs is required to withhold canonical mRNA export factors, including Yra1p, allowing certain mRNAs to bypass the quality control system and undergo immediate export mediated by Mex67p alone.

Taken together, our data indicate that Sgf73p orchestrates the selective and efficient export of target genes in response to specific environmental cues, and that this occurs through a direct interaction with various mRNA export factors.

## Discussion

**Fig. 7:**
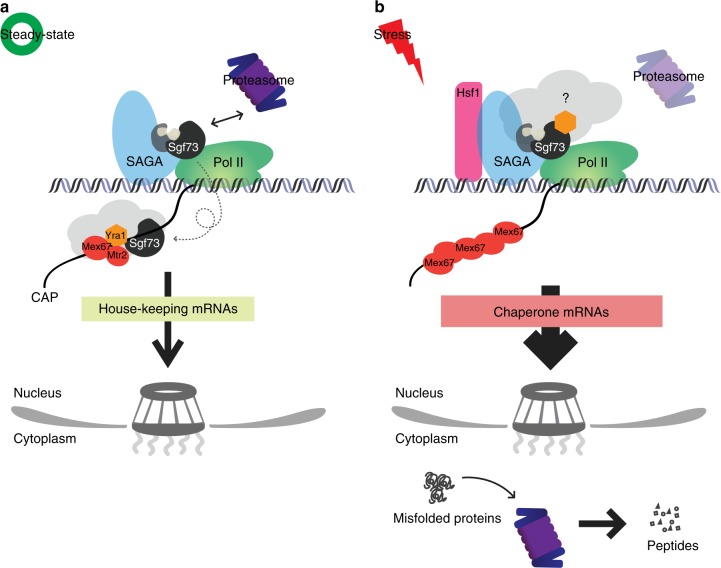
Sgf73p-mediated mRNP surveillance facilitates proteostasis. **a** Under physiological conditions, the proteasome-dependent separation of the Sgf73-DUBm from SAGA is required for surveillance of mRNP biogenesis. The physical interaction between Sgf73p and Yra1p facilitates proper chromatin recruitment and RNA binding of Yra1p. **b** During stress, Sgf73p accumulates in the UASs of specific genes, and Sgf73p retention initiates the Mex67p-mediated non-canonical mRNA export pathway to bypass quality control. Overall, Sgf73p regulates the plasticity of gene expression by interacting with key mRNA export factors to maintain proteostasis and maximize cell survival under environmental uncertainty

In this report, we show that Sgf73p of the SAGA deubiquitylating module directly interacts with the essential mRNA export factor, Yra1p, and is required for its proper chromatin localization and binding of nascent RNA. Under physiological conditions, Sgf73p, in conjunction with the central surveillance factor Rrp6p, oversees the nucleocytoplasmic translocation of export-competent mRNPs. Upon receipt of an environmental stimulus, such as heat shock stress or galactose induction, Sgf73p initiates the non-canonical mRNA export pathway, which is subject to minimal quality control, and facilitates the prompt export of selective gene sets (Fig. 7). Thus, Sgf73p-mediated switching between the canonical and non-canonical mRNA export pathways orchestrates the plasticity of gene expression to maximize cell survival.

mRNA export is a tightly coordinated process during which various factors are co-transcriptionally recruited to the transcription site and nascent RNA. Previous studies have shown that the RNA polymerase II (RNAPII) CTD-bound Pcf11p is responsible for recruiting Yra1p to the transcription site. Our findings here show that Sgf73p physically interacts with Yra1p, but not with Pcf11p. Additionally, our in vitro competition assay indicates that Pcf11p and Sgf73p competes in binding Yra1p. Based on these findings, we prefer the model in which Yra1p is initially recruited to chromatin by Pcf11p and is handed-over to Sgf73p for further processes of mRNA export. However, further investigation is needed to fully describe the complex network among these factors, including a more comprehensive analysis of the chromatin localization.

The SAGA complex is composed of four distinct modules, two of which have enzymatic activities: the histone acetyltransferase module and the histone deubiquitylating module (DUBm). SAGA was initially shown to be important for both transcriptional activation and elongation^41,42^, and recent work has highlighted its role in mRNA export, especially through the DUBm^12,15^. Here, we report direct evidence showing that SAGA contributes to the biogenesis quality control and export of mRNPs through the DUBm subunit, Sgf73p. Our findings strongly support the notion that SAGA intricately coordinates gene expression from the activation step through elongation and export. We believe that the arrangement of functional modules within SAGA serves as an efficient platform for the proteasome to regulate proteostasis and effectively respond to environmental stress.

In the last decade, it was widely accepted that there was a clear distinction between the SAGA-dependent and TFIID-dependent gene sets, and that SAGA and TFIID specifically regulated stress-related and housekeeping genes, respectively^28^. However, recent studies have questioned the accuracy of this classification^26,30^. In an effort to newly define genes that are directly regulated by the SAGA complex, we herein identified the Sgf73-peak gene set. This gene set did not show any prevalent bias towards either SAGA-dependent or TFIID-dependent genes, indicating that the regulatory jurisdiction of SAGA is not confined to the previously defined gene set. Instead, we propose that the SAGA complex executes a more complicated and dynamic mode of gene expression regulation.

The proteasome is believed to confer non-proteolytic functions through its chaperone-like activity. For example, the 19S RP of the proteasome has been demonstrated to stimulate promoter targeting of co-activators^43^ and contribute to transcriptional elongation through an interaction with the FACT complex^44,45^. We previously showed that a physical interaction between the 19S RP and Sgf73p of SAGA is critical for proper mRNA export^10^. Here, we report that, upon environmental stress (e.g., heat shock and oxidative stress), Sgf73p selectively occupies the UASs of molecular chaperone transcripts, which are required for cell survival during stress^46^. Given that the release of Sgf73-DUBm from the SAGA complex is facilitated by the 19S RP, our findings suggest that there is a close relationship between the proteolytic and non-proteolytic functions of the proteasome, both of which contribute to proteostasis (Fig. 7).

In agreement with our findings, starvation in yeast was reported to induce up-regulation of Hsf1p targets^47^. The *GAL1* gene encodes the Gal1p galactokinase, which catalyzes the conversion of α-d-galactose to galactose-1-phosphate; in the absence of Gal1p, galactose catabolism is perturbed and cells display a proteotoxic phenotype^48^. Thus, consistent with cells under heat shock and oxidative stress, cells grown in galactose-based medium require the concerted function of Sgf73p and the proteasome for survival. Altogether, our findings here demonstrate a comprehensive role of the proteasome in protein homeostasis regulation.

## Methods

### Yeast strains and plasmid construction

The strains and plasmid constructs used in this study are detailed in Supplementary Tables 1 and 2, respectively. Genomic integration of C-terminal tags and gene deletions were performed by homologous recombination of PCR fragments^49^. Cells were cultured in yeast peptone dextrose (YPD) medium at 30 °C unless otherwise indicated. For galactose induction assays, cells were cultured in YP + 2% raffinose medium to mid-log phase and an additional 2% galactose was added. Plasmid constructs were prepared using an EZ-Fusion Cloning Kit (Enzynomics, EZ015).

### Spotting assay

Cells were spotted onto the indicated plates with a starting OD of 0.5 and five-fold serial dilutions, unless otherwise noted. Plates were incubated at the indicated temperatures for ~2–3 days.

### Co-immunoprecipitation

Cells were cultured to mid-log phase, harvested by centrifugation (total of 20OD), resuspended in NP40 lysis buffer [1% NP40, 150 mM NaCl, 2 mM EDTA, 6 mM Na_2_HPO_4_, 4 mM NaH_2_PO_4_, and protease inhibitors] and lysed by bead beating at 4 °C. Cell lysates were centrifuged at 13,000 rpm for 20 min at 4 °C, mixed with IgG or M2 beads (GE Healthcare, 17-0969-01, and Sigma, A2220, respectively) and incubated at 4 °C for 2 h with agitation. The beads were washed three times with 1 ml NP40 lysis buffer, and the bound proteins were eluted by boiling at 100 °C for 5 min in 2X SDS sample buffer and quantified by Western blotting (anti-MYC antibody; in-house; 1:3000, anti-FLAG M2 antibody; Sigma; F1804; 1:3000). Uncropped Western-blots can be found in Supplementary Figs. 10 and  11.

### Affinity purification

For TAP-tagged protein purification, yeast cells were grown in YPD at 30 °C to an OD of ~1.0, harvested, resuspended in Workman buffer [40 mM HEPES–KOH (pH 7.5), 350 mM NaCl, 10% glycerol, 0.1% Tween-20, and protease inhibitors] and lysed using a mixer mill (Retsch, MM400). Cell extracts were subjected to a brief centrifugation and the collected supernatants were applied to IgG Sepharose (GE Healthcare, 17-0969-01) for 3 h, and then to 5 µg TEV overnight at 4 °C. The TEV-treated supernatants were mixed with calmodulin sepharose (Stratagene, 214303) in Calmodulin-binding buffer [10 mM Tris–HCl (pH 8.0), 1 mM MgAc, 1 mM imidazole, 2 mM CaCl_2_, 0.1% NP40, 10 mM β-mercaptoethanol, 300 mM KCl, 10% glycerol, and protease inhibitors]. Bound proteins were eluted in Calmodulin elution buffer [10 mM Tris–HCl (pH 8.0), 150 mM NaCl, 1 mM MgAc, 1 mM imidazole, 3 mM EGTA, 0.1% NP40, 10% glycerol, and protease inhibitors] and analyzed by Western blotting (anti-CBP antibody; in-house: 1:3000).

### In vitro protein-pulldown assay

Bait protein (1 µg) and 200 ng target protein were mixed with 50 µl Pulldown buffer [50 mM HEPES–KOH (pH 7.5), 150 mM NaCl, 10% glycerol, 0.1% NP40, 0.5 mM DTT, 1 mM EDTA, and protease inhibitors] and 10 µl of beads, and incubated at 4 °C for 2 h. The protein-bound beads were washed three times with 1 ml Pulldown buffer. The bound proteins were eluted by boiling at 100 °C for 5 min in 20 µl 2X SDS sample buffer, and quantified by Western blotting (anti-HA antibody; in-house; 1:3000, anti-FLAG M2 antibody; Sigma; F1804; 1:3000, anti-MBP antibody; in-house; 1:3000).

### Fluorescence in situ hybridization and imaging

Cells were cultured to early log-phase (0.2OD) and pre-fixed in 0.1 volume of 37% formaldehyde for 15 min at room temperature. The cells were harvested by centrifugation and fixed in 10 ml 4% para-formaldehyde (Alfa Aesar) in phosphate buffer for 3 h at room temperature with agitation. The cells were briefly washed twice using 10 ml ice-cold Solution B [1.2 M sorbitol and 0.1 M KPO_4_] and resuspended in 500 µl Zymolase B [Solution B and 0.2% β-mercaptoethanol] and 50 µl 10 mg/ml 20T Zymolase (USBiological) to prepare spheroplasts. Permeabilized cells were washed using 1 ml ice-cold Solution B, and applied to poly-l-lysine (Sigma)-coated cover slips. The cells were then treated with pre-hybridization B [10% dextran sulfate, 0.2% BSA, 0.3 M NaCl, 30 mM trisodium citrate dehydrate, 125 µg/ml yeast tRNA, 500 µg/ml denatured sonicated ssDNA, and 0.1 U/µl RNasin] and incubated in a humid chamber at 37 °C for 1 h. After being washed with 2X SSC B [0.3 M NaCl and 30 mM trisodium citrate dehydrate], the cells were treated with Hybridization B [Pre-hybridization B plus 1 ng/ml Cy3-labeled oligo dT (50) probe] and incubated in a humid chamber at 37 °C overnight. Nuclei were counterstained using 25 ng/ml 4′,6-diamidino-2-phenylindole (DAPI). After two brief washes using distilled water, the cover slips were mounted onto slide glasses. Microscopy was performed using an Imager M1 fluorescence microscope equipped with an AxioCam HRm camera and the Axio Vision 4.3 software (Zeiss). Images were acquired using a ×100 oil immersion lens and filter sets 20 (488020-0000) and 49 (488049-0000) for Cy3 and DAPI signals, respectively (Zeiss). For smFISH, probes were designed against the GFP sequence as previously described^40^ (LGC Biosearch Technologies) and images were acquired using an Andor Zyla 4.2 sCMOS camera. Signal intensities were quantified using the ImageJ software^50^.

### Chromatin immunoprecipitation

Cells were cultured under appropriate conditions and fixed with 1% (final concentration) formaldehyde for 15 min at room temperature with shaking. Fixation was stopped by the addition of glycine (final concentration, 125 mM). Cells were harvested by centrifugation, washed once using ice-cold 1X TBS buffer [20 mM Tris–HCl (pH 7.5) and 150 mM NaCl], and lysed in FA-M2 lysis buffer [50 mM Tris–HCl (pH 7.5), 150 mM NaCl, 1 mM EDTA and 1% Triton X-100] with bead beating at 4 °C. Supernatants were collected and subjected to sonication (Branson, 550) for chromatin shearing. The sonication conditions were optimized to obtain fragment lengths of 200–500 bp. The sonicated extracts were centrifuged at 13,000 rpm for 20 min at 4 °C, and the supernatants were incubated with 1 µg of anti-FLAG M2 antibody (Sigma, F1804, 1 mg/ml) and 20 µl Protein G Sepharose (GE Healthcare, 17-0618-50) at 4 °C overnight with rotation. After stringent washes, the immunoprecipitated chromatin was eluted using ChIP elution buffer [50 mM Tris–HCl (pH 8.0), 10 mM EDTA, and 1% SDS] at 65 °C with agitation. Eluted and input DNA were treated with RNase A (10 µg for 1 h at 37 °C; Roche, 1010916900) and proteinase K (60 µg for 2 h at 55 °C; Promega, V3021), and decrosslinked at 65 °C overnight. The final ChIP DNA was purified using a QIAquick PCR Purification Kit (QIAGEN, 28106). Quantitative analyses were performed using a CFX96 Real-Time System (Bio-Rad, C1000 Thermal Cycler). The utilized primers are listed in Supplementary Table 3.

### ChIP-seq library preparation and data analysis

ChIP-seq libraries were prepared using a NEXTflex ChIP-Seq Kit (Bioo Scientific Corporation, 5143-02) according to the manufacturer’s protocol. Sequencing was performed on a HiSeq2500 platform (single-end, 50 bp). Sequenced reads were mapped to the *S. cerevisiae* genome (sacCer3) and processed using Bowtie2^51^ and SAMtools^52^. Significant peaks were identified and annotated using HOMER^25^. Quantification analyses and average plots were generated using DeepTools^53^.

### RNA-immunoprecipitation

Cells cultured at appropriate conditions were fixed with 1% (final concentration) formaldehyde for 15 min at room temperature with shaking. Fixation was stopped by the addition of glycine (final concentration, 125 mM). Cells were harvested by centrifugation, washed once using ice-cold 1X TBS buffer [20 mM Tris–HCl (pH 7.5) and 150 mM NaCl], and lysed in FA-M2 lysis buffer [50 mM Tris–HCl (pH 7.5), 150 mM NaCl, 1 mM EDTA, 1% Triton X-100 and 40 U/ml RNasin] with bead beating at 4 °C. Cell extracts were subjected to sonication and centrifugation at 13,000 rpm for 20 min at 4 °C, and 500 µg of supernatant was mixed with 10 µl Dynabeads Protein G (Invitrogen, 10003D) and 1 µg anti-FLAG antibody or anti-mouse IgG (Sigma, F1804 and Millipore, 12-371, respectively) in a final volume of 500 µl, and incubated at 4 °C for 2 h. After stringent washes, bound RNA was eluted in FA-M2 elution buffer [50 mM Tris–HCl (pH 7.5), 10 mM EDTA, 1% SDS, and 40 U/ml RNasin] by incubation at 37 °C for 20 min with agitation. Eluted RNA and input samples were treated with proteinase K and decrosslinked at 65 °C for 2 h. RNA was purified using an RNeasy MiniElute Cleanup Kit (QIAGEN, 74204). cDNA was prepared by reverse transcription using random hexamers, and quantified using a CFX96 Real-Time System (Bio-Rad, C1000 Thermal Cycler).

### In vitro RNA-pulldown assay

*GAL1* RNA was prepared by in vitro transcription using a TranscriptAid T7 High Yield Transcription Kit (Thermo, K0441). The pulldown mix was prepared in 500 µl in vitro pulldown buffer [40 mM Tris–HCl (pH 8.0), 150 mM NaCl, 0.5 mM MgAc, 1 mM DTT, 0.01% NP40, 5% glycerol, 0.5% BSA, 100 µg/ml yeast tRNA, 0.1 U/µl RNasin and protease inhibitors] and contained 1 µg target protein, 500 ng in vitro-transcribed RNA, Dynabeads Protein A (Invitrogen, 10001D) and 2.5 µg antibody (anti-MBP, in-house, 1:3000). The pulldown mix was incubated at 25 °C for 2 h and washed five times with 1 ml in vitro pulldown buffer. Washed beads were resuspended in 100 µl in vitro pulldown buffer, and 10 µl was collected for Western blotting analysis. Bound RNA was purified by PCIAA treatment and the ethanol-down method. cDNA was prepared by reverse transcription using random hexamers, and quantified using a CFX96 Real-Time System (Bio-Rad, C1000 Thermal Cycler).

### RNA electrophoretic mobility shift assay

*GAL1* RNA was prepared using a TranscriptAid T7 High Yield Transcription kit (K0441, Thermo Scientific) and biotinylated using a Pierce RNA 3′ End Biotinylation kit (20160, Thermo Scientific) according to the manufacturer’s protocol. Gel-purified biotinylated *GAL1* RNA was used for RNA-EMSA. The indicated amount of MBP alone or MBP-Sgf73p was incubated with 10 nM of biotinylated GAL1 RNA in binding buffer (50 mM Tris–HCl (pH 7.5), 100 mM NaCl, 10 mM β-mercaptoethanol, 5% glycerol) for 40 min at room temperature with gentle agitation. RNA–protein complexes were immobilized on 5% native gel and transferred to an Amersham Hybond+N membrane (RPN303B, GE Healthcare). After a brief UV crosslinking, signals were visualized using a Chemiluminescent Nucleic Acid Detection Module (89880, Thermo Scientific) according to the manufacturer’s protocol.

## Supplementary information

Supplementary Information

Source Data

## Data Availability

All sequencing data that support the findings of this study have been deposited in the National Center for Biotechnology Information Gene Expression Omnibus (GEO; http://www.ncbi.nlm.nih.gov/geo/) and are accessible through the GEO Series accession number GSE116228. All other relevant data are available from the corresponding author upon request.

## References

[1] Chavez S (2000). A protein complex containing Tho2, Hpr1, Mft1 and a novel protein, Thp2, connects transcription elongation with mitotic recombination in *Saccharomyces cerevisiae*. EMBO J..

[2] Strasser K (2002). TREX is a conserved complex coupling transcription with messenger RNA export. Nature.

[3] Stutz F (2000). REF, an evolutionary conserved family of hnRNP-like proteins, interacts with TAP/Mex67p and participates in mRNA nuclear export. RNA.

[4] Zenklusen D, Vinciguerra P, Strahm Y, Stutz F (2001). The yeast hnRNP-Like proteins Yra1p and Yra2p participate in mRNA export through interaction with Mex67p. Mol. Cell Biol..

[5] Segref A (1997). Mex67p, a novel factor for nuclear mRNA export, binds to both poly(A) + RNA and nuclear pores. EMBO J..

[6] Fischer T (2002). The mRNA export machinery requires the novel Sac3p-Thp1p complex to dock at the nucleoplasmic entrance of the nuclear pores. EMBO J..

[7] Mitchell P, Petfalski E, Shevchenko A, Mann M, Tollervey D (1997). The exosome: a conserved eukaryotic RNA processing complex containing multiple 3′–>5′ exoribonucleases. Cell.

[8] Hilleren P, McCarthy T, Rosbash M, Parker R, Jensen TH (2001). Quality control of mRNA 3′-end processing is linked to the nuclear exosome. Nature.

[9] Iglesias N (2010). Ubiquitin-mediated mRNP dynamics and surveillance prior to budding yeast mRNA export. Genes Dev..

[10] Lim S, Kwak J, Kim M, Lee D (2013). Separation of a functional deubiquitylating module from the SAGA complex by the proteasome regulatory particle. Nat. Commun..

[11] Strasser K, Hurt E (2000). Yra1p, a conserved nuclear RNA-binding protein, interacts directly with Mex67p and is required for mRNA export. EMBO J..

[12] Rodriguez-Navarro S (2004). Sus1, a functional component of the SAGA histone acetylase complex and the nuclear pore-associated mRNA export machinery. Cell.

[13] Iglesias N, Stutz F (2008). Regulation of mRNP dynamics along the export pathway. FEBS Lett..

[14] Pascual-Garcia P (2008). Sus1 is recruited to coding regions and functions during transcription elongation in association with SAGA and TREX2. Genes Dev..

[15] Kohler A, Schneider M, Cabal GG, Nehrbass U, Hurt E (2008). Yeast Ataxin-7 links histone deubiquitination with gene gating and mRNA export. Nat. Cell Biol..

[16] Johnson SA, Cubberley G, Bentley DL (2009). Cotranscriptional recruitment of the mRNA export factor Yra1 by direct interaction with the 3′ end processing factor Pcf11. Mol. Cell.

[17] Amrani N (1997). PCF11 encodes a third protein component of yeast cleavage and polyadenylation factor I. Mol. Cell Biol..

[18] Collins SR (2007). Functional dissection of protein complexes involved in yeast chromosome biology using a genetic interaction map. Nature.

[19] Zenklusen D, Vinciguerra P, Wyss JC, Stutz F (2002). Stable mRNP formation and export require cotranscriptional recruitment of the mRNA export factors Yra1p and Sub2p by Hpr1p. Mol. Cell Biol..

[20] Canavan R, Bond U (2007). Deletion of the nuclear exosome component RRP6 leads to continued accumulation of the histone mRNA HTB1 in S-phase of the cell cycle in *Saccharomyces cerevisiae*. Nucleic Acids Res..

[21] Babour A (2016). The chromatin remodeler ISW1 is a quality control factor that surveys nuclear mRNP biogenesis. Cell.

[22] Torchet C (2002). Processing of 3′-extended read-through transcripts by the exosome can generate functional mRNAs. Mol. Cell.

[23] Jensen TH (2004). Modulation of transcription affects mRNP quality. Mol. Cell.

[24] Haruki H, Nishikawa J, Laemmli UK (2008). The anchor-away technique: rapid, conditional establishment of yeast mutant phenotypes. Mol. Cell.

[25] Heinz S (2010). Simple combinations of lineage-determining transcription factors prime cis-regulatory elements required for macrophage and B cell identities. Mol. Cell.

[26] Baptista Tiago, Grünberg Sebastian, Minoungou Nadège, Koster Maria J.E., Timmers H.T. Marc, Hahn Steve, Devys Didier, Tora László (2017). SAGA Is a General Cofactor for RNA Polymerase II Transcription. Molecular Cell.

[27] Jeronimo C, Robert F (2014). Kin28 regulates the transient association of mediator with core promoters. Nat. Struct. Mol. Biol..

[28] Huisinga KL, Pugh BF (2004). A genome-wide housekeeping role for TFIID and a highly regulated stress-related role for SAGA in *Saccharomyces cerevisiae*. Mol. Cell.

[29] Lee TI (2000). Redundant roles for the TFIID and SAGA complexes in global transcription. Nature.

[30] Bonnet J (2014). The SAGA coactivator complex acts on the whole transcribed genome and is required for RNA polymerase II transcription. Genes Dev..

[31] Johnson SA, Kim H, Erickson B, Bentley DL (2011). The export factor Yra1 modulates mRNA 3′ end processing. Nat. Struct. Mol. Biol..

[32] Gavalda S, Santos-Pereira JM, Garcia-Rubio ML, Luna R, Aguilera A (2016). Excess of Yra1 RNA-binding factor causes transcription-dependent genome instability, replication impairment and telomere shortening. PLoS Genet..

[33] Meinel DM (2013). Recruitment of TREX to the transcription machinery by its direct binding to the phospho-CTD of RNA polymerase II. PLoS Genet..

[34] Hurt E (2000). Mex67p mediates nuclear export of a variety of RNA polymerase II transcripts. J. Biol. Chem..

[35] Solis EJ (2016). Defining the essential function of yeast Hsf1 reveals a compact transcriptional program for maintaining eukaryotic proteostasis. Mol. Cell.

[36] Yamamoto A, Ueda J, Yamamoto N, Hashikawa N, Sakurai H (2007). Role of heat shock transcription factor in *Saccharomyces cerevisiae* oxidative stress response. Eukaryot. Cell.

[37] Kaganovich D, Kopito R, Frydman J (2008). Misfolded proteins partition between two distinct quality control compartments. Nature.

[38] Escusa-Toret S, Vonk WI, Frydman J (2013). Spatial sequestration of misfolded proteins by a dynamic chaperone pathway enhances cellular fitness during stress. Nat. Cell Biol..

[39] Ghislain M, Udvardy A, Mann C (1993). *S. cerevisiae* 26S protease mutants arrest cell division in G2/metaphase. Nature.

[40] Zander Gesa, Hackmann Alexandra, Bender Lysann, Becker Daniel, Lingner Thomas, Salinas Gabriela, Krebber Heike (2016). mRNA quality control is bypassed for immediate export of stress-responsive transcripts. Nature.

[41] Grant PA (1998). A subset of TAF(II)s are integral components of the SAGA complex required for nucleosome acetylation and transcriptional stimulation. Cell.

[42] Henry KW (2003). Transcriptional activation via sequential histone H2B ubiquitylation and deubiquitylation, mediated by SAGA-associated Ubp8. Genes Dev..

[43] Lee D (2005). The proteasome regulatory particle alters the SAGA coactivator to enhance its interactions with transcriptional activators. Cell.

[44] Ferdous A, Gonzalez F, Sun L, Kodadek T, Johnston SA (2001). The 19S regulatory particle of the proteasome is required for efficient transcription elongation by RNA polymerase II. Mol. Cell.

[45] Sun L, Johnston SA, Kodadek T (2002). Physical association of the APIS complex and general transcription factors. Biochem. Biophys. Res. Commun..

[46] Feder ME, Hofmann GE (1999). Heat-shock proteins, molecular chaperones, and the stress response: evolutionary and ecological physiology. Annu. Rev. Physiol..

[47] Zid BM, O’Shea EK (2014). Promoter sequences direct cytoplasmic localization and translation of mRNAs during starvation in yeast. Nature.

[48] De Robichon-Szulmajster H (1958). Induction of enzymes of the galactose pathway in mutants of *Saccharomyces cerevisiae*. Science.

[49] Longtine MS (1998). Additional modules for versatile and economical PCR-based gene deletion and modification in *Saccharomyces cerevisiae*. Yeast.

[50] Schneider CA, Rasband WS, Eliceiri KW (2012). NIH Image to ImageJ: 25 years of image analysis. Nat. Methods.

[51] Langmead B, Trapnell C, Pop M, Salzberg SL (2009). Ultrafast and memory-efficient alignment of short DNA sequences to the human genome. Genome Biol..

[52] Li H (2009). The sequence Alignment/Map format and SAMtools. Bioinformatics.

[53] Ramirez F (2016). deepTools2: a next generation web server for deep-sequencing data analysis. Nucleic Acids Res..

