# Supplementary material for: SAGA DUBm-mediated surveillance regulates prompt export of stress-inducible transcripts for proteostasis
Source: Nat Commun. 2019 Jun 5;10:2458. doi: 10.1038/s41467-019-10350-6 (PMC6549176; doi:10.1038/s41467-019-10350-6)
Supplement: Supplementary file 1 — Supplementary Information [file 41467_2019_10350_MOESM1_ESM.pdf]

Supplementary Information

SAGA DUBm-mediated surveillance regulates prompt export of stress-inducible transcripts for proteostasis

Kim et al.

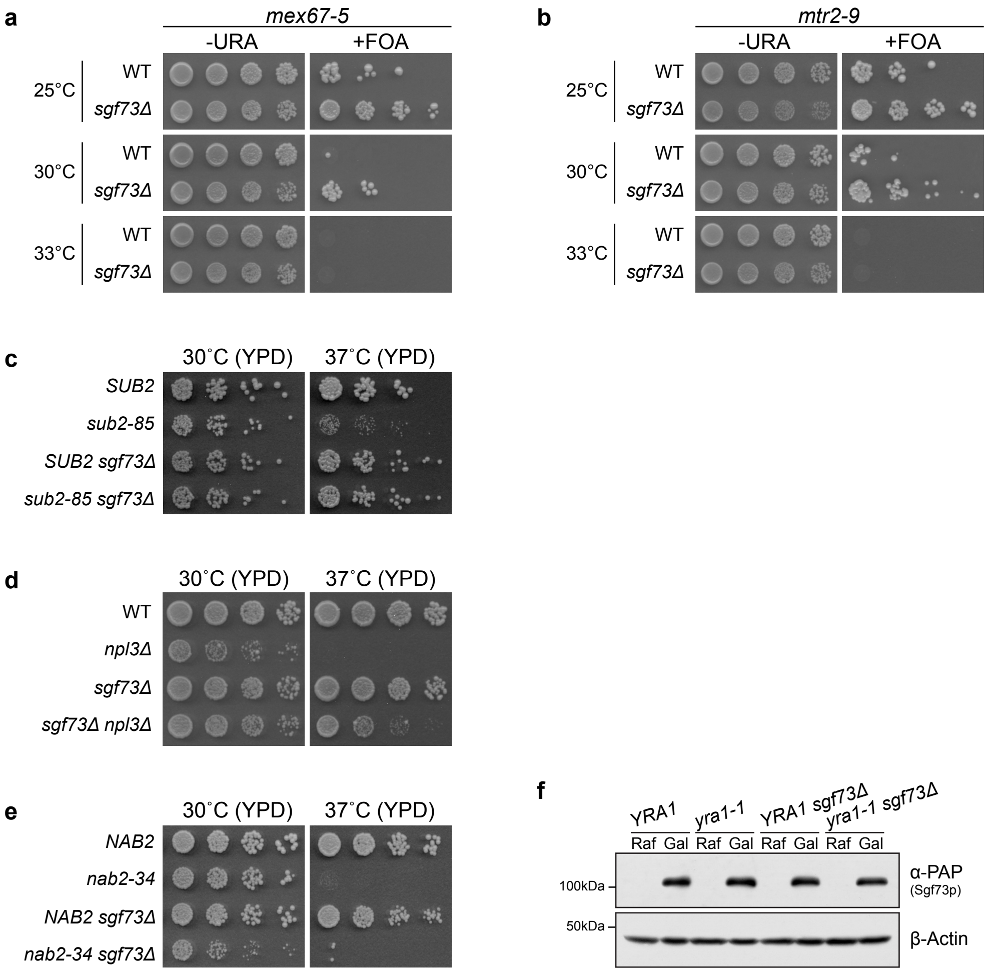

Supplementary Figure 1 *SGF73* deletion restores the growth defects of various mRNA export-impaired mutants. **a** and **b** The *ts*-mutant alleles, *mex67-5* (**a**) and *mtr2-9* (**b**), were transformed into wildtype and *sgf73Δ* cells carrying *MEX67* and *MTR2* shuffle vectors. Cells were plated in 5-fold dilutions onto SD-URA medium with or without 5'-fluoroorotic acid (-URA and +FOA, respectively) to shuffle out the *MEX67* and *MTR2* vectors. Plates were incubated at 25˚C, 30˚C and 37˚C for 3 days. **c**, **d** and **e** Spotting assays were performed using double mutants of *sgf73Δ* and (**c**) *sub2-85*, (**d**) *npl3Δ* and (**e**) *nab2-34*. Cells were plated onto YPD medium in 5-fold serial dilutions and incubated at 30˚C and 37˚C. **f** Western blot analysis of Sgf73p (α-PAP) over-expression in *yra1-ts* mutants. Cells were cultured in YP + 2% raffinose medium to mid-log phase, and an additional 2% galactose was added to induce Sgf73p over-expression. β-Actin and Ponceau staining were detected as loading controls.

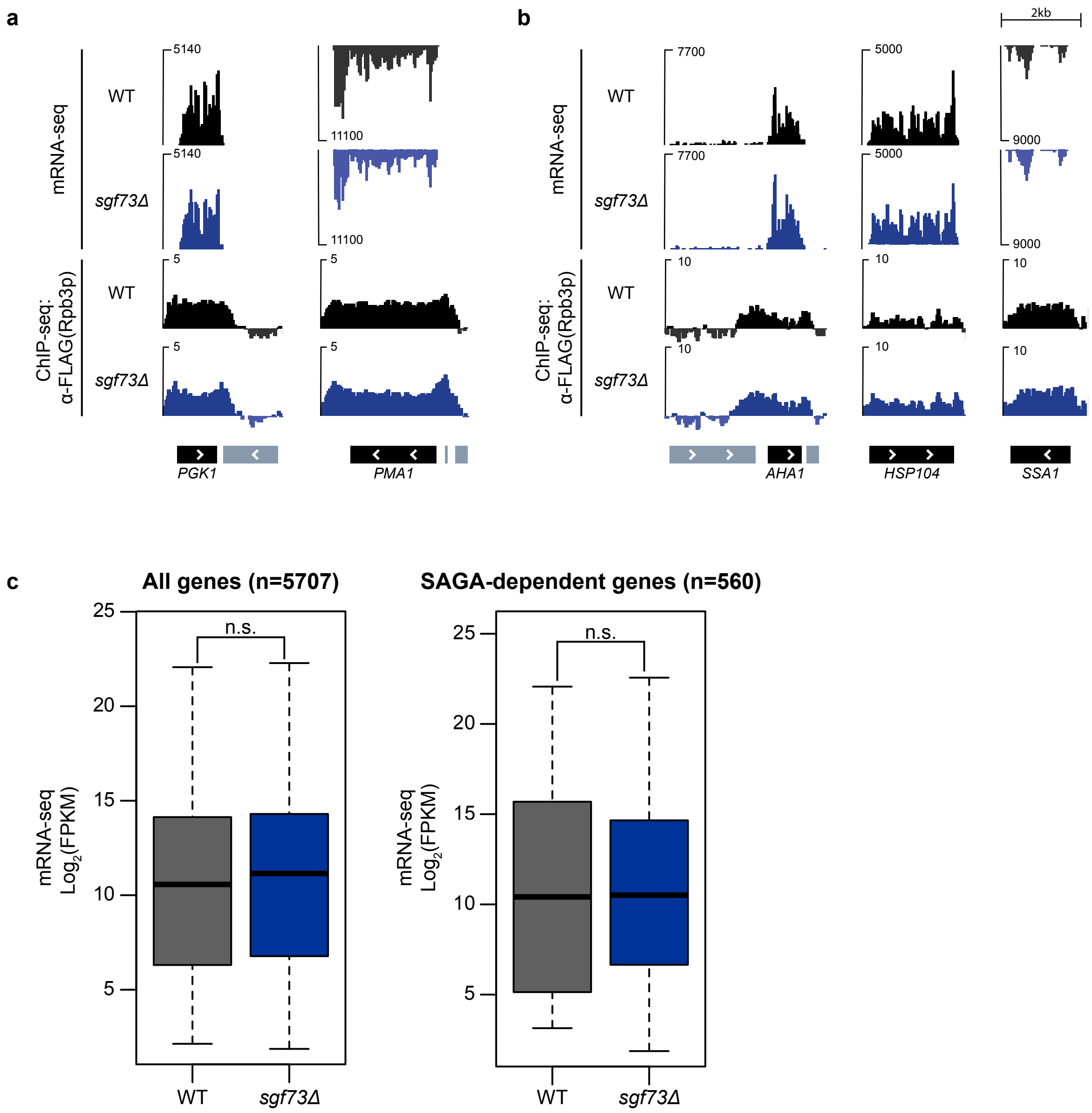

Supplementary Figure 2 Gene expression pattern of wildtype and the *sgf73*Δ mutant. **a** and **b** Genome browser view of mRNA-seq and RNAPII ChIP-seq (α-Rpb3) data regarding (**a**) Sgf73-peak genes *PGK1* and *PMA1* and (**b**) stress-inducible genes *AHA1*, *HSP104* and *SSA1* genes after heat shock. **c** Boxplot of Log2(FPKM) values from mRNA-seq data of wildtype and *sgf73Δ* cells regarding all genes (left panel) and SAGA-dependent genes (right panel).

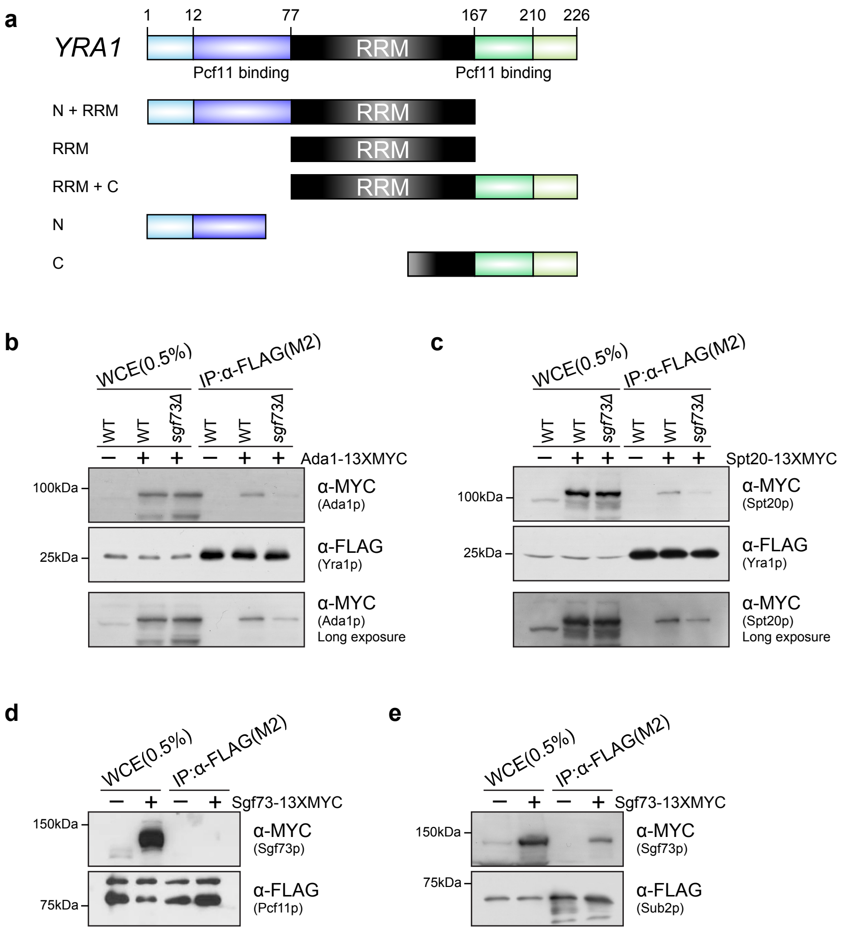

Supplementary Figure 3 The binding of Yra1p with SAGA is significantly reduced in the *sgf73Δ* mutant. **a** Domain diagrams of full-length and the subfragments of Yra1p used in the *in vitro* pulldown assays. **b** and **c** Co-IP assays of Yra1p (α-FLAG) and the SAGA subunits, Ada1p (α-MYC) (**b**) and Spt20p (α-MYC) (**c**), in wildtype and *sgf73Δ* cells. **d** and **e** Co-IP of (**d**) Pcf11p (α-FLAG) and Sgf73p (α-MYC) and (**e**) Sub2p (α-FLAG) and Sgf73p (α-MYC) in wildtype cells.

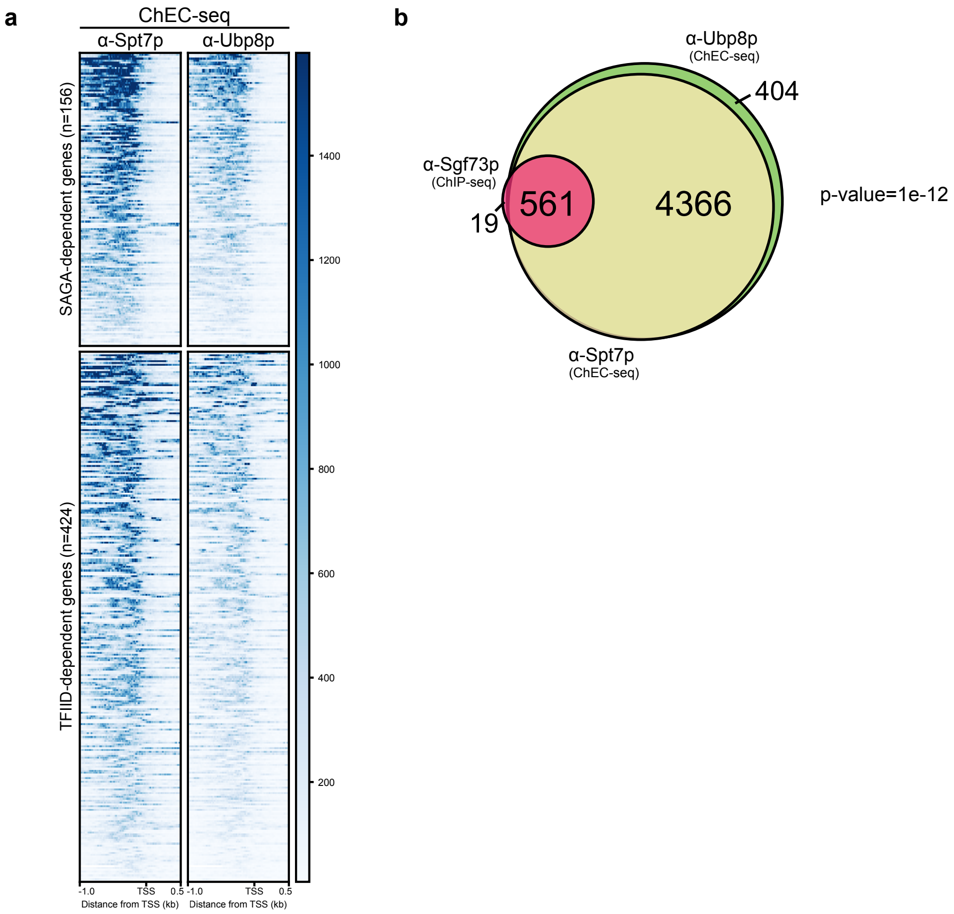

Supplementary Figure 4 Identification of Sgf73p-retained gene set. **a** Heatmaps of ChEC-seq signals representing SAGA binding (α-Spt7p and α-Ubp8p) in wildtype cells 1 kb upstream of the TSS, relative to the Sgf73-peak gene set (n=580). Heatmaps were sorted in descending order of Spt7p enrichment. The y-axis color scale indicates normalized ChEC-seq read count. **b** Venn diagram of genes that overlap (highlighted in red) between the Sgf73-peak and SAGA-occupied gene sets. kb, kilobase. TSS, transcription start site. P-value was calculated using the hypergeometric distribution.

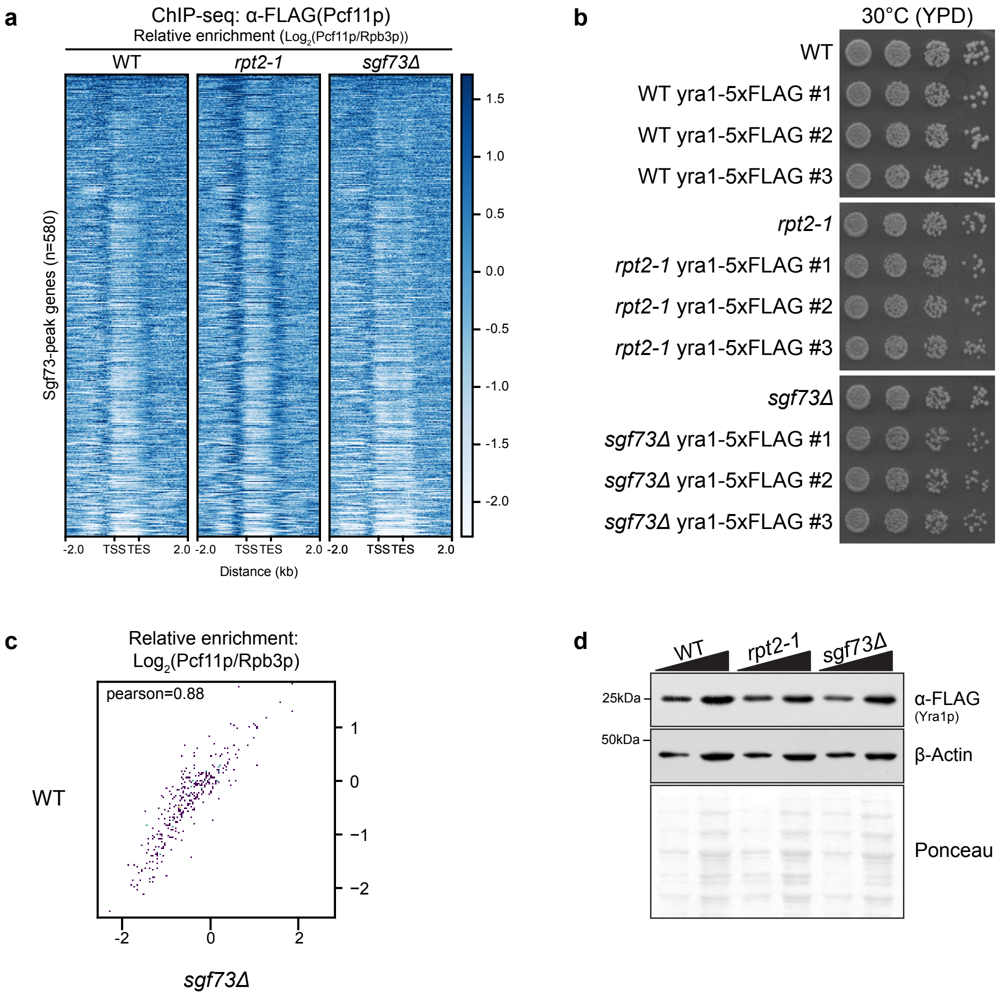

Supplementary Figure 5 5xFLAG tagging of Yra1p does not affect cell growth in wildtype, *rpt2-1* and *sgf73Δ* cells. **a** Heatmap of ChIP-seq signals representing Pcf11-5xFLAG enrichment in wildtype and *sgf73Δ* cells with respect to the Sgf73-peak gene set (n=580). **b** Spotting assays of Yra1p-5xFLAG-tagged wildtype, *rpt2-1* and *sgf73Δ* cells. Cells were plated on YPD medium in 5-fold serial dilutions and incubated at 30˚C. **d** Western blot analysis of Yra1p-5xFLAG expression levels in wildtype, *rpt2-1* and *sgf73Δ* cells. β-Actin and Ponceau staining were detected as loading controls.

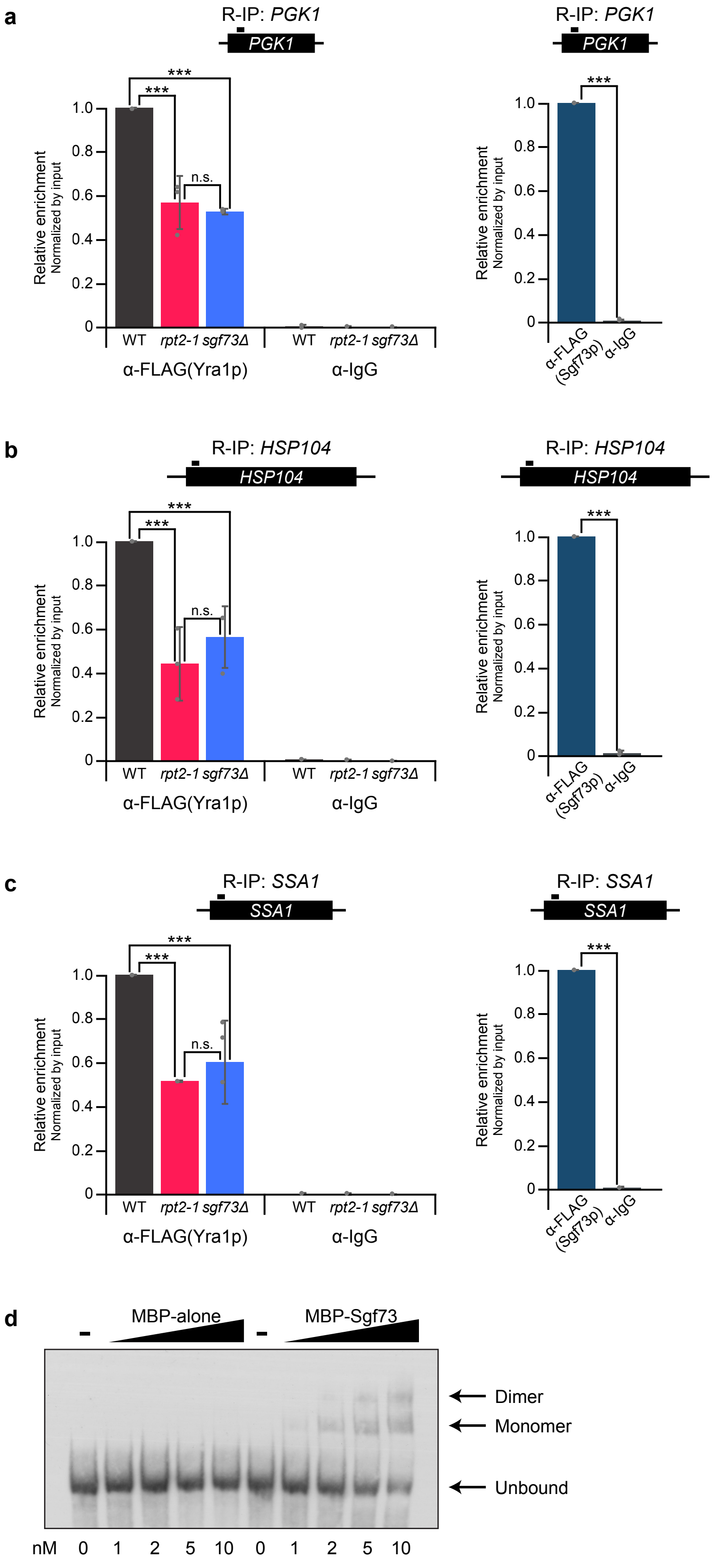

Supplementary Figure 6 RNA binding of Yra1p is reduced in *rpt2-1* and *sgf73Δ* mutants. **a**, **b** and **c** R-IP assays of Yra1p (α-FLAG) in wildtype, *rpt2-1* and *sgf73Δ* cells and of Sgf73p (α-FLAG) in wildtype cells, with respect to *PGK1*, *HSP104* and *SSA1* RNA transcripts. **d** RNA-EMSA of MBP-alone and MBP-Sgf73 using biotinylated *GAL1* transcript. Standard deviations of three independent experiments are shown by error bars and p-values were determined by Student's t-test (*P ≤ 0.05, **P ≤ 0.01, and ***P ≤ 0.001).

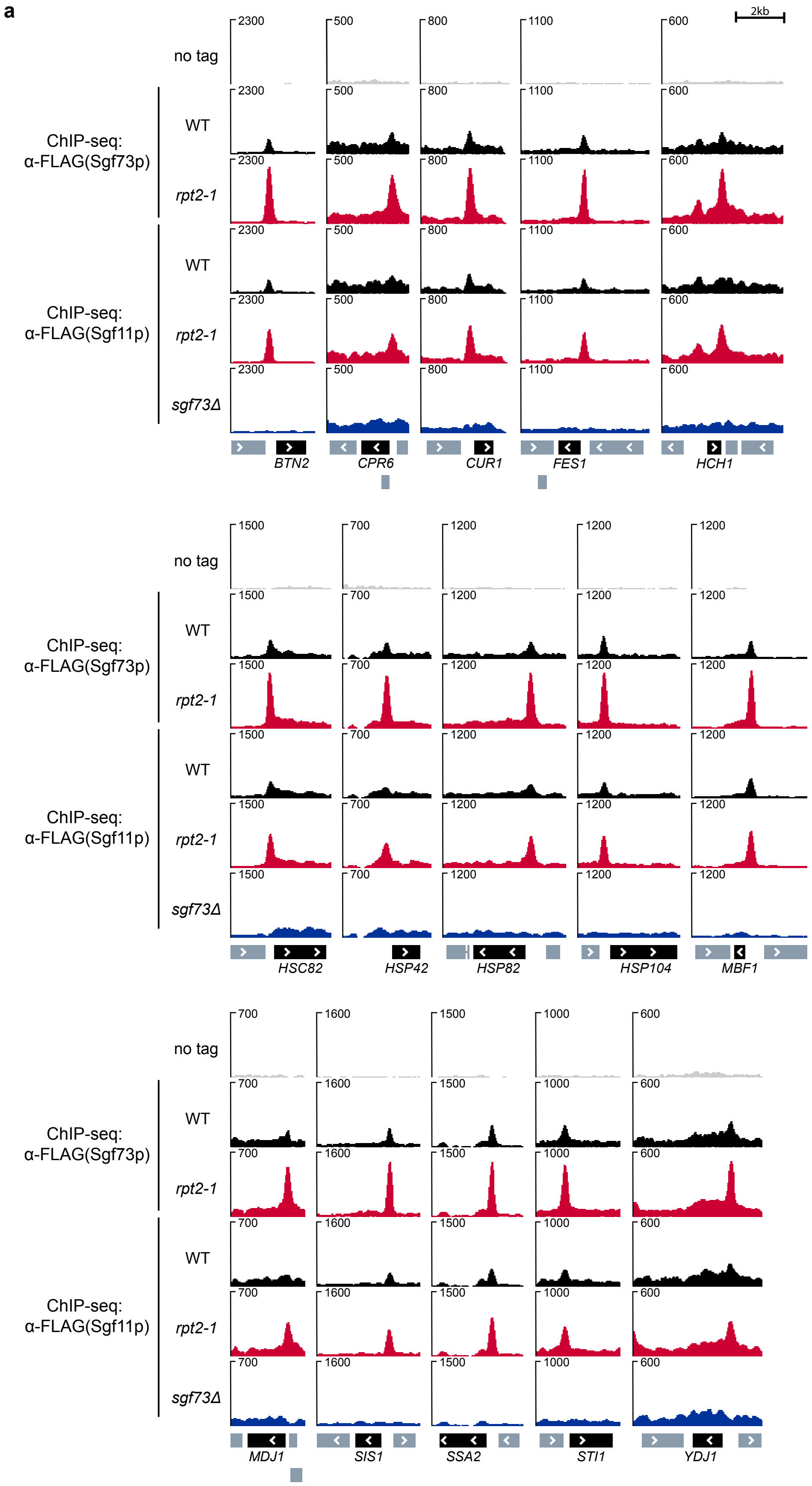

Supplementary Figure 7 Sgf73p is retained in the UAS elements of Hsf1-dependent genes. **a** Genome browser view of Sgf73p retention in the UAS elements of Hsf1-dependent genes (n=18).

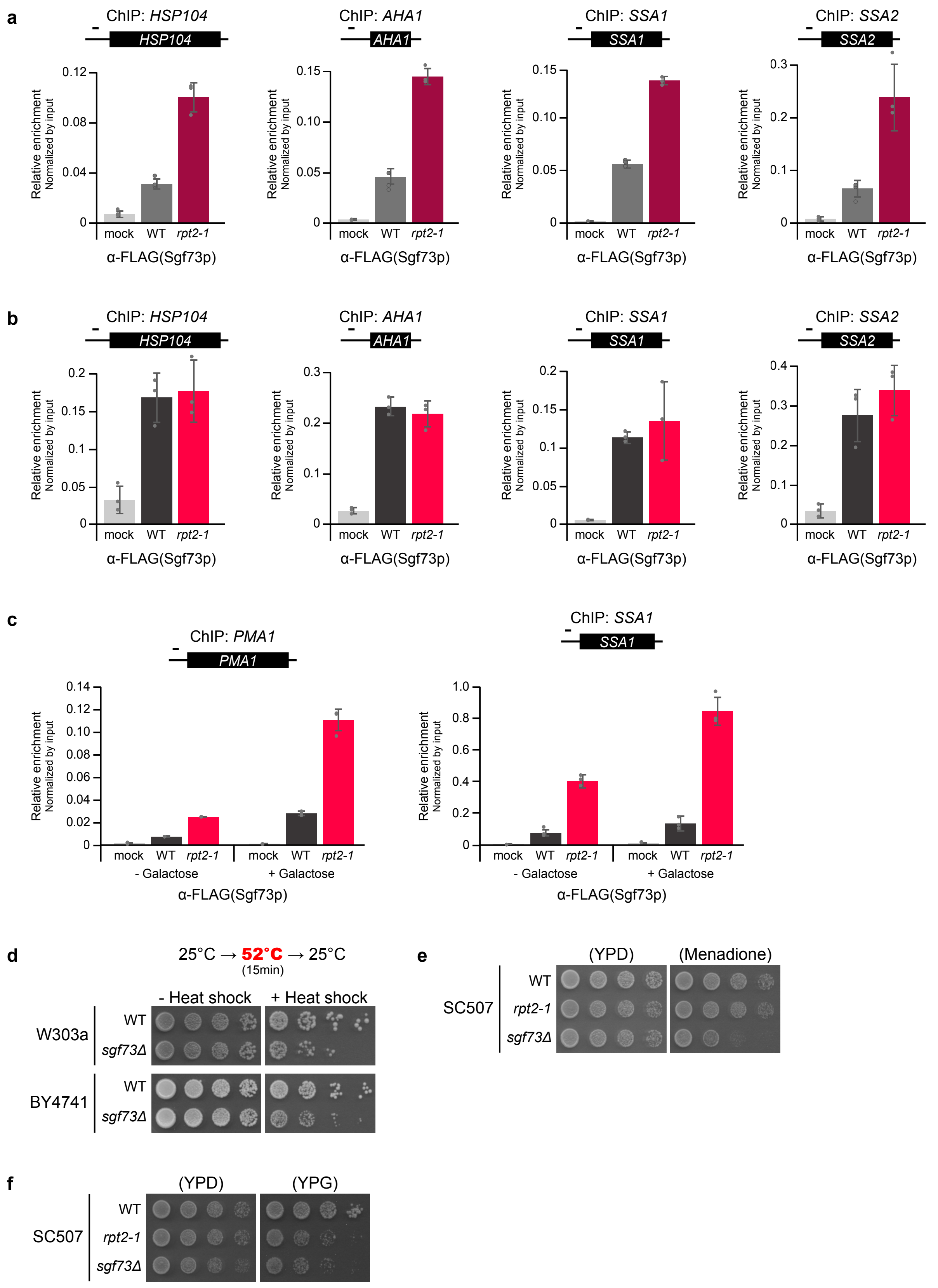
a
ChIP: HSP104
ChIP: AHA1
ChIP: SSA1
ChIP: SSA2
Relative enrichment
Normalized by input
mock
WT
rpt2-1
α-FLAG(Sgf73p)
b
c
ChIP: PMA1
- Galactose
+ Galactose
d
25°C → 52°C → 25°C
(15min)
- Heat shock
+ Heat shock
W303a
BY4741
WT
sgf73Δ
e
(YPD)
(Menadione)
SC507
f
(YPG)

Supplementary Figure 8 Sgf73p is required for cell survival after heat shock. **a** and **b** ChIP-qPCR against Sgf73-5xFLAG was performed in wildtype and *rpt2-1* cells (**a**) without and (**b**) with oxidative stress. **c** ChIP-qPCR against Sgf73-5xFLAG was performed in mock, wildtype and *rpt2-1* cells before and after galactose induction regarding *PMA1* and *SSA1* genes. ChIP-qPCR assays were performed on three independent extracts and standard deviations are shown by error bars. **d** *sgf73Δ* mutants of two different isogenic backgrounds, W303a and BY4741, were subjected to severe heat shock at 52˚C for 15 min, plated on YPD medium in 5-fold serial dilutions, and incubated at 25˚C for 3 days. **e** Oxidative stress tolerance was assessed by spotting assays. Wildtype, *rpt2-1* and *sgf73Δ* cells were plated on YPD and Menadione (20 nM) plates in 5-fold serial dilutions and incubated at 25˚C for 3 days. **f** Cell growth was assessed in galactose-based medium. Wildtype, *rpt2-1* and *sgf73Δ* cells were plated on YPD and YP + 2% raffinose medium and incubated for 3 days.

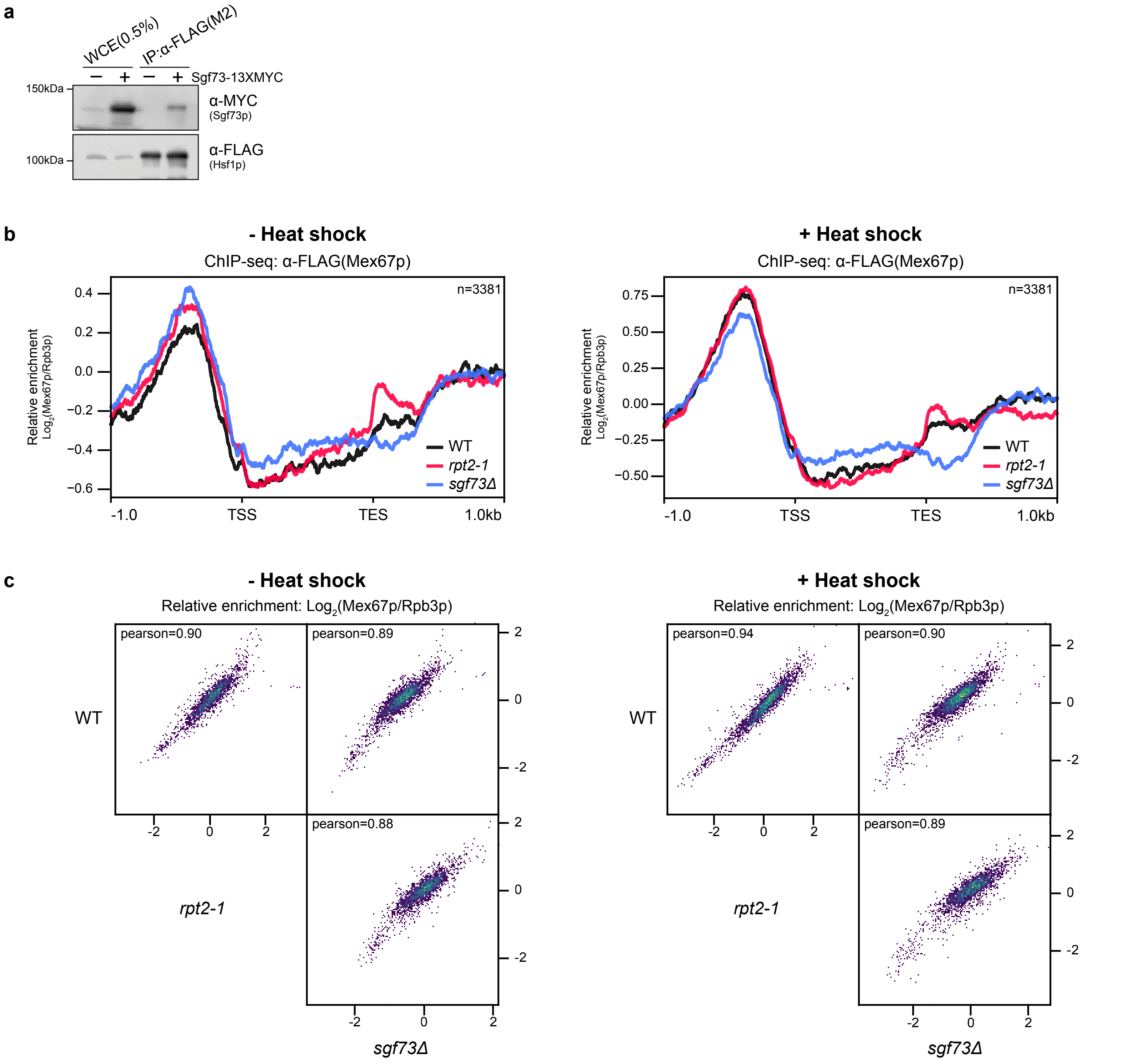

Supplementary Figure 9 Enrichment of Mex67p is minimally affected in non-induced genes in the *sgf73*Δ mutant. **a** Co-IP of Hsf1p (α-FLAG) and Sgf73p (α-MYC) in wildtype cells. **b** Average plot of Mex67-5xFLAG enrichment in wildtype, *rpt2-1* and *sgf73Δ* cells with respect to the non-Hsf1-dependent gene set (n=3381) before and after heat shock. **c** Scatterplots showing the correlation between Mex67p ChIP-seq signals of wildtype, *rpt2-1* and *sgf73*Δ mutants regarding non-Hsf1p genes. Correlation coefficients were calculated by the Pearson method.

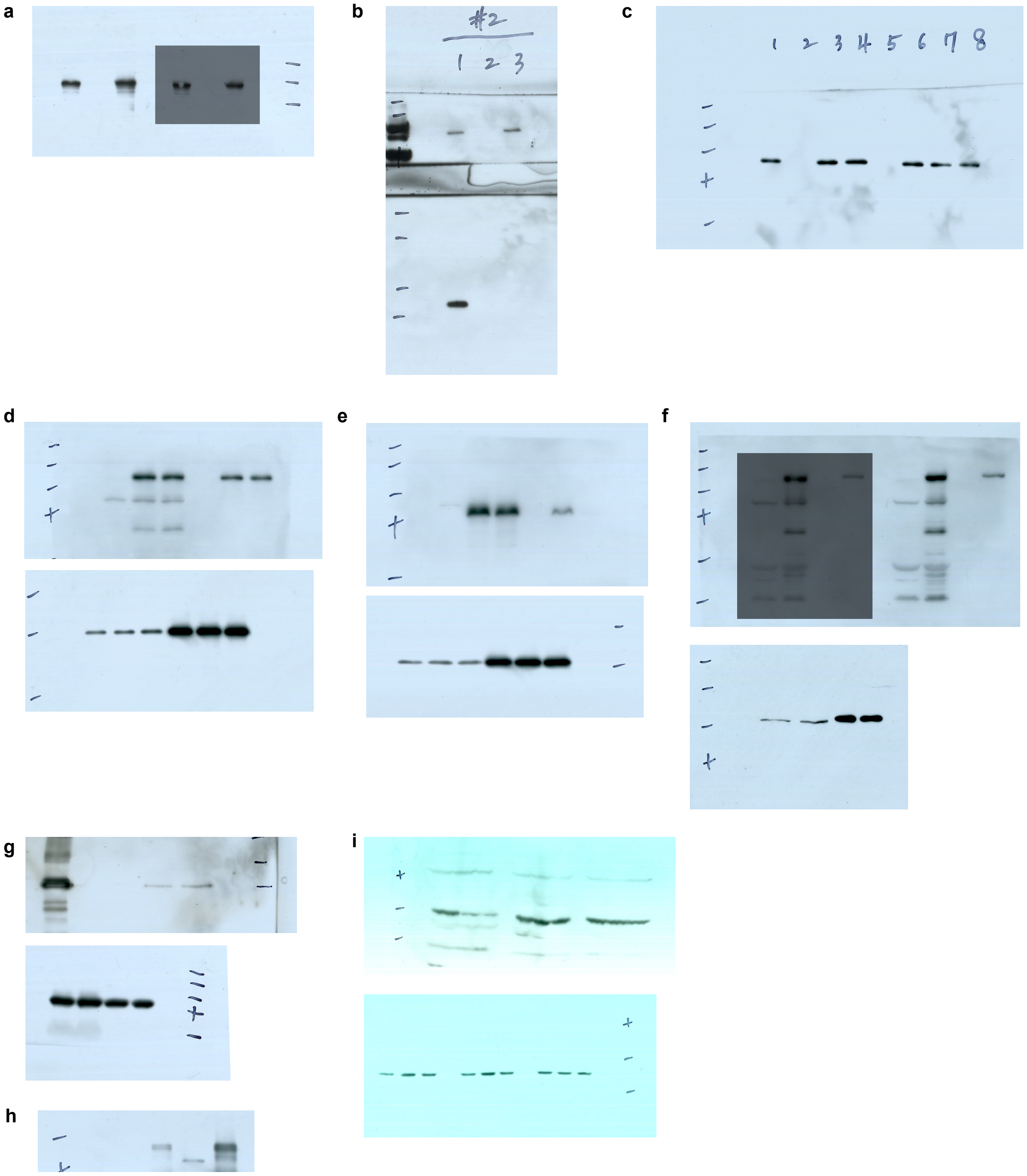

Supplementary Figure 10 Original, uncropped Western blot film in Figures. The uncropped scans of Western blot exposed to X-ray files in Figures **a** Fig. 1c, **b** Fig. 1d, **c** Fig. 1e, **d** Fig. 1f, **e** Fig. 1g, **f** Fig. 2b, **g** Fig. 4d, **h** Fig. 4g and **i** Fig. 5g.

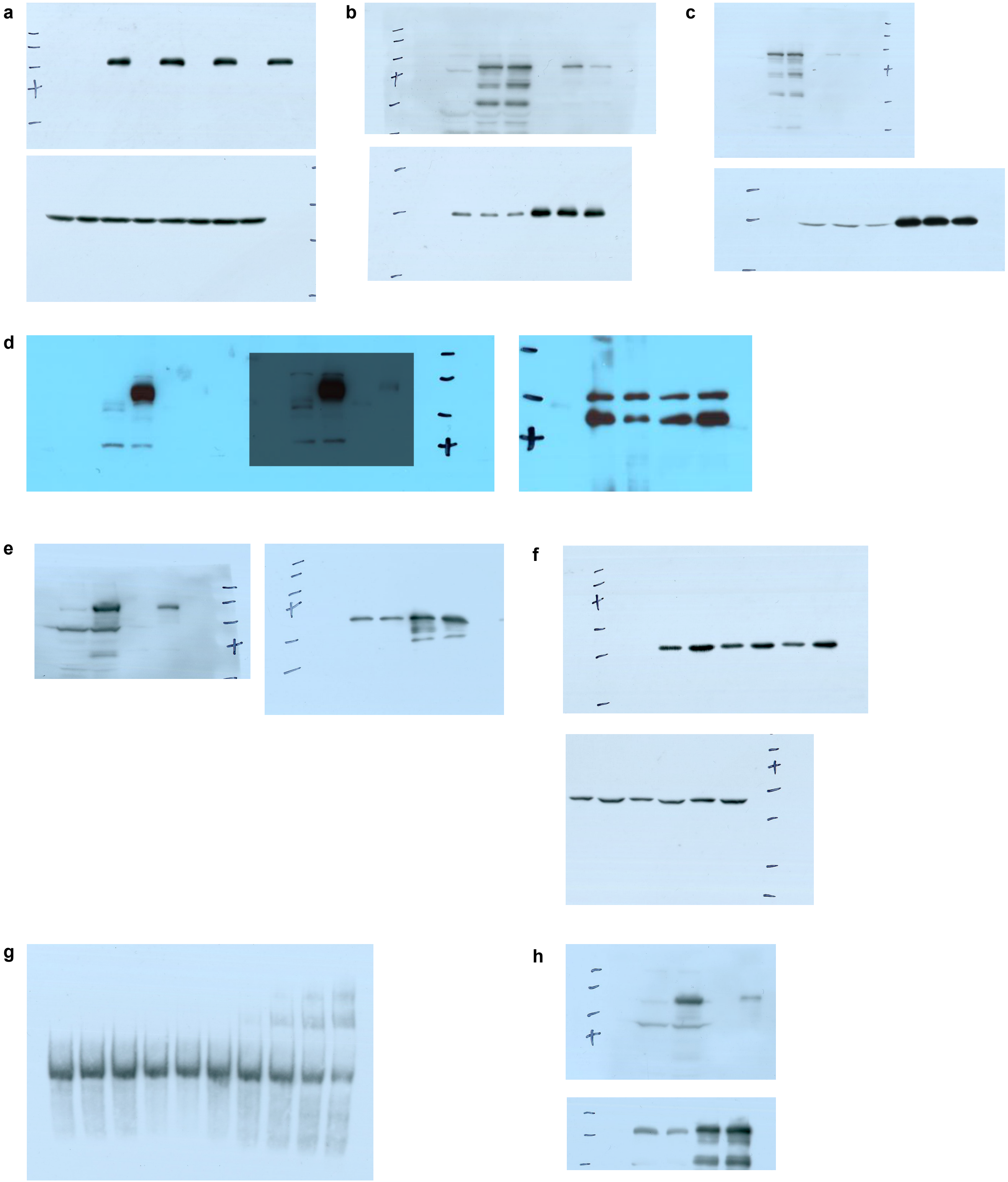

Supplementary Figure 11 Original, uncropped Western blot film in Supplementary Figures. The uncropped scans of Western blot exposed to X-ray files in Supplementary Figures **a** 1f, **b** 3b, **c** 3c, **d** 3d, **e** 3e, **f** 5c, **g** 6d and **h** 9a.

Supplementary Table 1 List of strains used in this study

#	Yeast strain	Genotype	Reference
1	ySS501	MATa, can1-100, ade2-1, his3-11, -15, leu2-3, -112, trp1-1, ura3-1	Lee, D. *et al.* (2005)
2	ySS106	MATa, can1-100, ade2-1, his3-11, -15, leu2-3, -112, trp1-1, ura3-1, RPT2ΔRKKRK	Lim, S. *et al.* (2013)
3	yMK052	MATa, can1-100, ade2-1, his3-11, -15, leu2-3, -112, trp1-1, ura3-1, sgf73Δ::natNT2	This study
4	yMK168	MATa, can1-100, ade2-1, his3-11, -15, leu2-3, -112, trp1-1, ura3-1, SGF73-5xFLAG::HYG	This study
5	yMK179	MATa, can1-100, ade2-1, his3-11, -15, leu2-3, -112, trp1-1, ura3-1, RPT2ΔRKKRK, SGF73-5xFLAG::HYG	This study
6	SC048	MATa, can1-100, ade2-1, his3-11, -15, leu2-3, -112, trp1-1, ura3-1, SGF11-5xFLAG::HYG	This study
7	SC050	MATa, can1-100, ade2-1, his3-11, -15, leu2-3, -112, trp1-1, ura3-1, RPT2ΔRKKRK, SGF11-5xFLAG::HYG	This study
8	SC082	MATa, can1-100, ade2-1, his3-11, -15, leu2-3, -112, trp1-1, ura3-1, sgf73Δ::natNT2, SGF11-5xFLAG::HYG	This study
9	SC154	MATa, can1-100, ade2-1, his3-11, -15, leu2-3, -112, trp1-1, ura3-1, SUA7-5xFLAG::HYG	This study
10	SC158	MATa, can1-100, ade2-1, his3-11, -15, leu2-3, -112, trp1-1, ura3-1, RPT2ΔRKKRK, SUA7-5xFLAG::HYG	This study
11	SC161	MATa, can1-100, ade2-1, his3-11, -15, leu2-3, -112, trp1-1, ura3-1, sgf73Δ::natNT2, SUA7-5xFLAG::HYG	This study
12	SC170	MATa, can1-100, ade2-1, his3-11, -15, leu2-3, -112, trp1-1, ura3-1, RPB3-5xFLAG::HYG	This study
13	SC173	MATa, can1-100, ade2-1, his3-11, -15, leu2-3, -112, trp1-1, ura3-1, RPT2ΔRKKRK, RPB3-5xFLAG::HYG	This study
14	SC194	MATa, can1-100, ade2-1, his3-11, -15, leu2-3, -112, trp1-1, ura3-1, sgf73Δ::natNT2, RPB3-5xFLAG::HYG	This study
15	SC369	MATa, can1-100, ade2-1, his3-11, -15, leu2-3, -112, trp1-1, ura3-1, yra1Δ::natNT2, pRS316 YRA1	This study
16	SC371	MATa, can1-100, ade2-1, his3-11, -15, leu2-3, -112, trp1-1, ura3-1, yra1Δ::natNT2, pRS316 yra1-1	This study

17	SC446	MATa, can1-100, ade2-1, his3-11, -15, leu2-3, -112, trp1-1, ura3-1, sgf73Δ::HIS5, yra1Δ::natNT2, pRS316 YRA1	This study
18	SC447	MATa, can1-100, ade2-1, his3-11, -15, leu2-3, -112, trp1-1, ura3-1, sgf73Δ::HIS5, yra1Δ::natNT2, pRS316 yra1-1	This study
19	SC550	MATa, can1-100, ade2-1, his3-11, -15, leu2-3, -112, trp1-1, ura3-1, ubp8Δ::HIS5, yra1Δ::natNT2, pRS316 YRA1	This study
20	SC552	MATa, can1-100, ade2-1, his3-11, -15, leu2-3, -112, trp1-1, ura3-1, ubp8Δ::HIS5, yra1Δ::natNT2, pRS316 yra1-1	This study
21	SC555	MATa, can1-100, ade2-1, his3-11, -15, leu2-3, -112, trp1-1, ura3-1 sgf11Δ::HIS5, yra1Δ::natNT2 pRS316 YRA1	This study
22	SC557	MATa, can1-100, ade2-1, his3-11, -15, leu2-3, -112, trp1-1, ura3-1, sgf11Δ::HIS5, yra1Δ::natNT2, pRS316 yra1-1	This study
23	SC618	MATa, can1-100, ade2-1, his3-11, -15, leu2-3, -112, trp1-1, ura3-1, spt20Δ::HIS5, yra1Δ::natNT2, pRS316 YRA1	This study
24	SC621	MATa, can1-100, ade2-1, his3-11, -15, leu2-3, -112, trp1-1, ura3-1, spt20Δ::HIS5, yra1Δ::natNT2, pRS316 yra1-1	This study
25	SC608	MATa, can1-100, ade2-1, his3-11, -15, leu2-3, -112, trp1-1, ura3-1, gcn5Δ::HIS5, yra1Δ::natNT2, pRS316 YRA1	This study
26	SC610	MATa, can1-100, ade2-1, his3-11, -15, leu2-3, -112, trp1-1, ura3-1, gcn5Δ::HIS5, yra1Δ::natNT2, pRS316 yra1-1	This study
27	SC733	MATa, can1-100, ade2-1, his3-11, -15, leu2-3, -112, trp1-1, ura3-1, yra1Δ::natNT2, pRS316 YRA1, pGAL-SGF73-TAP::URA3	This study
28	SC735	MATa, can1-100, ade2-1, his3-11, -15, leu2-3, -112, trp1-1, ura3-1, yra1Δ::natNT2, pRS316 yra1-1, pGAL-SGF73-TAP::URA3	This study
29	SC741	MATa, can1-100, ade2-1, his3-11, -15, leu2-3, -112, trp1-1, ura3-1, sgf73Δ::HIS5, yra1Δ::natNT2, pRS316 YRA1, pGAL-SGF73-TAP::URA3	This study
30	SC743	MATa, can1-100, ade2-1, his3-11, -15, leu2-3, -112, trp1-1, ura3-1, sgf73Δ::HIS5, yra1Δ::natNT2, pRS316 yra1-1, pGAL-SGF73-TAP::URA3	This study
31	yMK203	MATa, can1-100, ade2-1, his3-11, -15, leu2-3, -112, trp1-1, ura3-1, YRA1-5xFLAG::HYG	This study

32	SC342	MATa, can1-100, ade2-1, his3-11, -15, leu2-3, -112, trp1-1, ura3-1, SGF73-13MYC::HIS5, YRA1-5xFLAG::HYG	This study
33	SC415	MATa, can1-100, ade2-1, his3-11, -15, leu2-3, -112, trp1-1, ura3-1, RPT2ΔRKKRK, SGF73-13MYC::HIS5, YRA1-5xFLAG::HYG	This study
34	SC468	MATa, can1-100, ade2-1, his3-11, -15, leu2-3, -112, trp1-1, ura3-1, UBP8-13MYC::HIS5, YRA1-5xFLAG::HYG	This study
35	SC503	MATa, can1-100, ade2-1, his3-11, -15, leu2-3, -112, trp1-1, ura3-1, sgf73Δ::natNT2, UBP8-13MYC::HIS5, YRA1-5xFLAG::HYG	This study
36	SC565	MATa, can1-100, ade2-1, his3-11, -15, leu2-3, -112, trp1-1, ura3-1, npl3Δ::HIS5	This study
37	SC587	MATa, can1-100, ade2-1, his3-11, -15, leu2-3, -112, trp1-1, ura3-1, sgf73Δ::kanMX6, npl3Δ::natNT2	This study
38	SC711	MATa, can1-100, ade2-1, his3-11, -15, leu2-3, -112, trp1-1, ura3-1, mtr2Δ::HIS5, pRS316 MTR2, pRS315 mtr2-9	This study
39	SC715	MATa, can1-100, ade2-1, his3-11, -15, leu2-3, -112, trp1-1, ura3-1, mtr2Δ::HIS5, sgf73Δ::natNT2, pRS316 MTR2, pRS315 mtr2-9	This study
40	SC705	MATa, can1-100, ade2-1, his3-11, -15, leu2-3, -112, trp1-1, ura3-1, mex67Δ::HIS5, pRS316 MEX67, pRS315 mex67-5	This study
41	SC709	MATa, can1-100, ade2-1, his3-11, -15, leu2-3, -112, trp1-1, ura3-1, mex67Δ::HIS5, sgf73Δ::natNT2, pRS316 MEX67, pRS315 mex67-5	This study
42	SC719	MATa, can1-100, ade2-1, his3-11, -15, leu2-3, -112, trp1-1, ura3-1, ADA1-13MYC::TRP1, YRA1-5xFLAG::HYG	This study
43	SC770	MATa, can1-100, ade2-1, his3-11, -15, leu2-3, -112, trp1-1, ura3-1, sgf73Δ::natNT2, ADA1-13MYC::TRP1, YRA1-5xFLAG::HYG	This study
44	SC723	MATa, can1-100, ade2-1, his3-11, -15, leu2-3, -112, trp1-1, ura3-1, SPT20-13MYC::kanMX6, YRA1-5xFLAG::HYG	This study
45	SC826	MATa, can1-100, ade2-1, his3-11, -15, leu2-3, -112, trp1-1, ura3-1, sgf73Δ::natNT2, SPT20-13MYC::HIS5, YRA1-5xFLAG::HYG	This study
46	SC058	MATa, can1-100, ade2-1, his3-11, -15, leu2-3, -112, trp1-1, ura3-1 PCF11-5xFLAG::HYG	This study
47	SC202	MATa, can1-100, ade2-1, his3-11, -15, leu2-3, -112, trp1-1, ura3-1, PCF11-5xFLAG::HYG, SGF73-13MYC::HIS	This study

48	SC043	MATa, can1-100, ade2-1, his3-11, -15, leu2-3, -112, trp1-1, ura3-1, rrp6Δ::natNT2	This study
49	SC062	MATa, can1-100, ade2-1, his3-11, -15, leu2-3, -112, trp1-1, ura3-1, sgf73Δ::natNT2, rrp6Δ::kanMX6	This study
50	yMK061	MATa, can1-100, ade2-1, his3-11, -15, leu2-3, -112, trp1-1, ura3-1, ubp8Δ::HIS5	This study
51	SC745	MATa, can1-100, ade2-1, his3-11, -15, leu2-3, -112, trp1-1, ura3-1, ubp8Δ::natNT2, rrp6Δ::kanMX6	This study
52	yMK024	MATa, can1-100, ade2-1, his3-11, -15, leu2-3, -112, trp1-1, ura3-1, sgf11Δ::natNT2	This study
53	SC747	MATa, can1-100, ade2-1, his3-11, -15, leu2-3, -112, trp1-1, ura3-1, sgf11Δ::natNT2, rrp6Δ::kanMX6	This study
54	yMK058	MATa, can1-100, ade2-1, his3-11, -15, leu2-3, -112, trp1-1, ura3-1, sus1Δ::trp1	This study
55	SC749	MATa, can1-100, ade2-1, his3-11, -15, leu2-3, -112, trp1-1, ura3-1, sus1Δ::natNT2, rrp6Δ::kanMX6	This study
56	SC263	MATa, can1-100, ade2-1, his3-11, -15, leu2-3, -112, trp1-1, ura3-1, gcn5Δ::natNT2	This study
57	SC751	MATa, can1-100, ade2-1, his3-11, -15, leu2-3, -112, trp1-1, ura3-1, gcn5Δ::natNT2, rrp6Δ::kanMX6	This study
58	SC281	MATa, can1-100, ade2-1, his3-11, -15, leu2-3, -112, trp1-1, ura3-1, spt20Δ::natNT2	This study
59	SC753	MATa, can1-100, ade2-1, his3-11, -15, leu2-3, -112, trp1-1, ura3-1, spt20Δ::natNT2, rrp6Δ::kanMX6	This study
60	SC828	MATa, can1-100, ade2-1, his3-11, -15, leu2-3, -112, trp1-1, ura3-1, RRP6-5xFLAG::HYG	This study
61	SC830	MATa, can1-100, ade2-1, his3-11, -15, leu2-3, -112, trp1-1, ura3-1, SGF73-13MYC::HIS5, RRP6-5xFLAG::HYG	This study
62	Y3515	MATalpha, rna14-3, ade2-1, leu2, his3-11, 15, ura3-1	Jensen, T. *et al.* (2004)
63	SC928	MATalpha, rna14-3, ade2-1, leu2, his3-11, 15, ura3-1, sgf73Δ::natNT2	This study
64	Y40343	MATalpha, ade2-1, trp1-1, can1-100, leu2-3,112, his3-11,15, ura3, GAL psi+, tor1-1, fpr1::NAT, RPL13A-2×FKBP12::TRP1	EUROSCARF
65	SC842	MATalpha, ade2-1, trp1-1, can1-100, leu2-3,112, his3-11,15, ura3, GAL psi+, tor1-1, fpr1::NAT, RPL13A-2×FKBP12::TRP1, RNA14-FRB-GFP::HIS	This study
66	SC846	MATalpha, ade2-1, trp1-1, can1-100, leu2-3,112, his3-11,15, ura3, GAL psi+, tor1-1, fpr1::NAT, RPL13A-2×FKBP12::TRP1, RNA14-FRB-GFP::HIS, sgf73Δ::kanMX6	This study

67	yMK206	MATa, can1-100, ade2-1, his3-11, -15, leu2-3, -112, trp1-1, ura3-1, RPT2ΔRKKRK, YRA1-5xFLAG::HYG	This study
68	yMK209	MATa, can1-100, ade2-1, his3-11, -15, leu2-3, -112, trp1-1, ura3-1, sgf73Δ::natNT2, YRA1-5xFLAG::HYG	This study
69	ySS510	MATalpha, ura3-52, leu2Δ1, cim3-1	Ghislain, M. *et al.* (1993)
70	SC920	MATalpha, ura3-52, leu2Δ1, cim3-1, pESC URA GFP ubc9ts	This study
71	SC916	MATa, can1-100, ade2-1, his3-11, -15, leu2-3, -112, trp1-1, ura3-1, pESC URA GFP ubc9ts	This study
72	SC918	MATa, can1-100, ade2-1, his3-11, -15, leu2-3, -112, trp1-1, ura3-1, sgf73Δ::natNT2, pESC URA GFP ubc9ts	This study
73	W303a	MATa, ade2-1, ura3-1, his3-11, trp1-1, leu2-3,112, can1-100	
74	yMK026	MATa, ade2-1, ura3-1, his3-11, trp1-1, leu2-3,112, can1-100, sgf73Δ::HIS5	This study
75	BY4741	MATa, his3D1, leu2D0, met15D0, ura3D0	
76	#4422	MATa, his3D1, leu2D0, met15D0, ura3D0, sgf73Δ::kanMX6	ThermoFisher
77	yMK211	MATa, can1-100, ade2-1, his3-11, -15, leu2-3, -112, trp1-1, ura3-1, MEX67-5xFLAG::HYG	This study
78	yMK214	MATa, can1-100, ade2-1, his3-11, -15, leu2-3, -112, trp1-1, ura3-1, RPT2ΔRKKRK, MEX67-5xFLAG::HYG	This study
79	yMK217	MATa, can1-100, ade2-1, his3-11, -15, leu2-3, -112, trp1-1, ura3-1, sgf73Δ::natNT2, MEX67-5xFLAG::HYG	This study
80	SC346	MATa, can1-100, ade2-1, his3-11, -15, leu2-3, -112, trp1-1, ura3-1, mlp1Δ::HIS5	This study
81	SC349	MATa, can1-100, ade2-1, his3-11, -15, leu2-3, -112, trp1-1, ura3-1, RPT2ΔRKKRK, mlp1Δ::HIS5	This study
82	SC350	MATa, can1-100, ade2-1, his3-11, -15, leu2-3, -112, trp1-1, ura3-1, sgf73Δ::natNT2, mlp1Δ::HIS5	This study
83	SC351	MATa, can1-100, ade2-1, his3-11, -15, leu2-3, -112, trp1-1, ura3-1, mlp2Δ::HIS5	This study
84	SC354	MATa, can1-100, ade2-1, his3-11, -15, leu2-3, -112, trp1-1, ura3-1, RPT2ΔRKKRK, mlp2Δ::HIS5	This study
85	SC357	MATa, can1-100, ade2-1, his3-11, -15, leu2-3, -112, trp1-1, ura3-1, sgf73Δ::natNT2, mlp2Δ::HIS5	This study

Supplementary Table 2 List of plasmid constructs used in this study

#	**Plasmid name**	**Contents**	**Reference**
1	pMH01	pRS314 YRA1	Strasser, K. & Hurt, E. (2000)
2	pMH02	pRS314 yra1-1	Strasser, K. & Hurt, E. (2000)
3	pMH03	pRS406 pGAL SGF73	This study
4	pMH04	pGEX4T-1 YRA1	This study
5	pMH05	pMAL-c2 SGF73	This study
6	pMH06	pRS316 MTR2	Santos-Rosa, H. *et al.* (1998)
7	pMH07	pRS315 mtr2-9	Santos-Rosa, H. *et al.* (1998)
8	pMH08	pRS316 MEX67	Segref, A. *et al.* (1997)
9	pMH09	pRS315 mex67-5	Segref, A. *et al.* (1997)
10	pMH10	pMAL-c2	
11	pMH11	pGEX4T-1 YRA1 N + RRM (1-167aa)	This study
12	pMH12	pGEX4T-1 YRA1 RRM (77-167aa)	This study
13	pMH13	pGEX4T-1 YRA1 RRM + C (77-226aa)	This study
14	pMH14	pGEX4T-1 YRA1 N (1-66aa)	This study
15	pMH15	pGEX4T-1 YRA1 N (124-226aa)	This study
16	pMH16	pET21(a)+ PCF11	This study
17	pMH17	pESC URA GFP ubc9ts	ADDGENE

Supplementary Table 3 List of plasmid constructs used in this study.

#	Name	Sequence
1	FISH_Oligo(dT)	5'-Cy3-oligo d(T)50-3'
2	HSP104_3'_F	GAAATTGAAGAGAGATTCGAGC
3	HSP104_3'_R	GATCCTTAGTGCCAGTTTGTTC
4	HSP104_RT_F	TTGAACTTGACACCCGAGCAA
5	HSP104_RT_R	AAGATTTGACGTCCAGTGGAC
6	PMA1_#2_F	CGACGACGAAGACAGTGATAACG
7	PMA1_#2_R	ATTGAATTGGACCGACGAAAAACATAAC
8	GAL1_#2_F	ATTTGCTCAAAGGAAGTTCGATT
9	GAL1_#2_R	GAAGACTTGCAGCCCG
10	GAL1_#1_F	CGCTTAACTGCTCATTGCTATATTG
11	GAL1_#1_R	TTG TTC GGA GCA GTG CGG CGC
12	pHSP12_F	CCCATTGACGTAGAAATTGAAAGAA
13	pHSP12_R	GAGTGCTTCTGGTCTGTGTT
14	pCYC1_F	CCGATCTTTCCGGTCTCTTTG
15	pCYC1_R	CCAAATGAACTGGCGCTTTG
16	PGK1_#2_F	AGCGTGTCTTCATCAGAG
17	PGK1_#2_R	TGGCAAAGCAGCAACAA
18	HSP104_#2_F	AGATCATAGTCGTAACGGC
19	HSP104_#2_R	ATATGAACGACCAAACGC
20	SSA1_#2_F	GATTGATTGGTGATGCTGCTAAG
21	SSA1_#2_R	TTCTGGGTCGTTGAAGTTTCT
22	HSP104_#1_F	GTCATCGATTCAAAGGCGTTATTC
23	HSP104_#1_R	GCCCTTGGAGTTTGGATTCT
24	AHA1_#1_F	GCCACAGAGGTCAAGGATAAA
25	AHA1_#1_R	CACCGAGGAAATCTAGCATCATA
26	SSA1_#1_F	TTTCCAGAACGTTCCATCGG
27	SSA1_#1_R	CTTGAAGTCCTCGAAACGATCA
28	SSA2_#1_F	ACTCTCTCTTGGGCCCTTAT
29	SSA2_#1_R	CTTCGAGAAAGTGAATCGGAAGA
30	PMA1_#1_F	GAAACGGAGAAACATAAACAGG
31	PMA1_#1_R	GTCTCGAGGCCTGGAAGTGC
